# A Reporter Platform to Study Therapy‐Induced Senescence in Live Cancer Cells

**DOI:** 10.1002/smtd.202501270

**Published:** 2025-10-29

**Authors:** Jacinta van de Grint, Mengqi Huang, Ruben Sangers, Hanny Odijk, Thom Reuvers, Jose M. Heredia‐Genestar, Anja Raams, Tsung Wai Kan, Joris Pothof, Roland Kanaar, Maayke M.P. Kuijten

**Affiliations:** ^1^ Department of Molecular Genetics Oncode Institute Erasmus MC Cancer Institute Erasmus University Medical Center Rotterdam 3015 GD the Netherlands; ^2^ Department of Pathology Erasmus University Medical Center Rotterdam 3015 GD the Netherlands

**Keywords:** DNA damage, lamins, live, monitoring, reporter, senescence

## Abstract

Senescence is a durable state of cell cycle arrest that can be induced by various stressors, including DNA damage caused by chemotherapeutic agents or ionizing radiation. In the context of cancer, therapy‐induced senescence (TIS) plays a dual role: while it effectively halts tumor cell proliferation, TIS also carries the risk of promoting tumor relapse through the senescence‐associated secretory phenotype (SASP). Beyond its direct impact on tumor cells, cancer therapies leading to TIS often induce short‐ and long‐term side effects that significantly affect the quality of life for patients. However, the lack of universal biomarkers for TIS hinders a comprehensive understanding of its characteristics and its role in cancer therapies. A lamin‐based senescence reporter platform is developed to reliably detect and sort live senescent cancer cells. This versatile tool supports live‐cell imaging, enabling real‐time tracking of senescence induction and escape to investigate heterogeneity in treatment response. Additionally, it allows high‐content screening and marker integration, for example incorporating IL6 as SASP marker. It is therefore a valuable tool for fundamental research addressing new questions in the field of TIS as well as for drug discovery, including the development of novel senolytics.

## Graphical abstract

This study describes the development and applications of a transgenic lamin‐based senescence reporter platform based on changes in lamin‐B1‐ and lamin‐A intensities during senescence‐induction. The reporter platform is a versatile tool, allowing to screen for and sort senescent cancer cells, allowing marker integration (for example incorporating IL6 upregulation as SASP marker) and enabling real‐time monitoring of senescence‐induction and ‐escape in cancer.

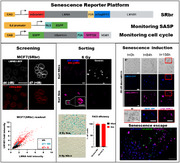

## 1 Introduction

Cellular senescence, a highly regulated form of cell‐cycle arrest, is mainly driven by the cyclin‐dependent kinase inhibitors p16^INK4a^, p15^INK4b^ and p21^CIP^ and is induced by various stress factors including oncogene activation and persistent DNA damage, induced for instance by critically short telomeres, ionizing radiation or chemotherapeutic drugs.^[^ ^1^, ^2^ ^]^ Senescent cells display multiple cellular and molecular features, including increased nuclear size, increased lysosomal content visualized by the presence of Senescence‐Associated Beta‐Galactosidase (SA‐β‐Gal) staining,^[^ ^2^, ^3^ ^]^ downregulation of proliferation markers, resistance to cell death, activation of the Senescence‐Associated Secretory Phenotype (SASP), widespread changes in chromatin organization and gene expression and often markers of DNA damage. Heterochromatin changes occurring in senescence include the formation of Senescence‐Associated Heterochromatin Foci (SAHF), enriched in tri‐methylation of Histone 3 lysine 9 (H3K9me3) present at sites of DNA damage^[^ ^4^, ^5^ ^]^ and a global reduction of H3K9me3 resulting in activation of promotor regions of SASP‐factors including interleukin 6 (IL6) and IL8.^[^ ^6^ ^]^ Lamins are nuclear membrane proteins involved in multiple cellular functions including cell division, DNA repair and chromatin organization and gene‐regulation.^[^ ^7^, ^8^ ^]^ In senescence lamin B1‐expression is reduced, which is acknowledged as a reliable biomarker.^[^ ^9^, ^10^ ^]^

Historically, senescence has been considered a tumor‐protective mechanism preventing uncontrolled proliferation. The effects on cell proliferation have been explored in anti‐cancer strategies in the form of therapy‐induced senescence (TIS), especially in combination with senolytics which clear senescent cells.^[^ ^11^, ^12^ ^]^ However, senescence also has a dark side: it contributes to organismal aging and to a plethora of age‐related degenerative diseases.^[^ ^13^, ^14^ ^]^ Moreover, the anti‐tumor barrier that senescence normally constitutes could be breached by senescence escape which could occur in multiple forms of senescence including TIS and oncogene‐induced senescence.^[^ ^15^, ^16^, ^17^ ^]^ In addition, TIS has been linked to tumor relapse, aggressiveness and metastasis.^[^ ^12^, ^18^ ^]^ These described contradictory effects of TIS emphasize the importance of monitoring senescence in cancer for instance in drug discovery trajectories. Unfortunately, screening for senescence‐inducing effects of compounds is hampered by the difficulty of identifying senescent cancer cells due to the lack of specific and universal biomarkers. To reap the beneficial effects of senescence while minimizing the detrimental effects, novel technologies are required to study the heterogeneous nature of senescence and gain understanding in characteristics of senescent cancer cells.

Here, we report on a senescence reporter platform for studying TIS in cancer cells. We developed transgenic lamin‐based reporters and utilized the changes in lamin B1‐ and lamin A intensities during senescence induction to screen and sort for senescent cells. Using the combination of the senescence reporters with senescence‐associated features, such as SASP, we revealed differences within senescent cell populations and between cancer models, relevant for understanding potential consequences of TIS in cancer. We show that with the senescence reporters we can monitor senescence induction and escape, gaining understanding of opportunities and potential risks of TIS as an anti‐cancer strategy. In summary, we show that our senescence reporter platform is a versatile tool to study TIS relevant for answering questions related to cancer therapies.

## 2 Results

### 2.1 Cancer‐Specific Lamina Remodeling During Senescence

Chemotherapy and irradiation are employed to kill cancer cells as these therapies induce DNA damage. However, this genotoxic stress can also lead to cellular senescence. TIS has emerged to be a pivotal cellular response in cancer treatment, as it is both contributing to therapeutic outcomes and potential long‐term side effects.^[^ ^19^ ^]^ To study the phenotypic characteristics of therapy‐induced senescent cancer cells, we primarily focused on two well‐characterized cancer cell lines that have previously been used to study TIS^[^ ^20^ ^]^: MCF7 (breast cancer; p53‐wild‐type, p16‐null)^[^ ^21^ ^]^ and A549 (lung cancer; p53‐wild‐type, p16‐null).^[^ ^22^ ^]^ We compared senescence induction using irradiation in these cancer cells to the induction in WI‐38, human lung fibroblasts (wild‐type p53/p16). Human diploid fibroblasts, such as WI‐38 and IMR90, have become classical models to study ageing. WI‐38 was originally described in the context of telomere shortening and is widely used to study senescence‐associated phenotypes.^[^ ^23^ ^]^ These cell models can undergo stress‐induced premature senescence (SIPS), for instance due to the induction of DNA damage via irradiation or chemotherapy. The phenotype of SIPS shares common features with replicative senescence including the classic markers: morphology, SA‐β‐Gal activity, cell cycle regulation and gene expression.^[^ ^24^ ^]^ Therefore, these models provide a robust framework to examine the classical features and mechanisms of senescence in response to genotoxic stress.

**Figure 1:**
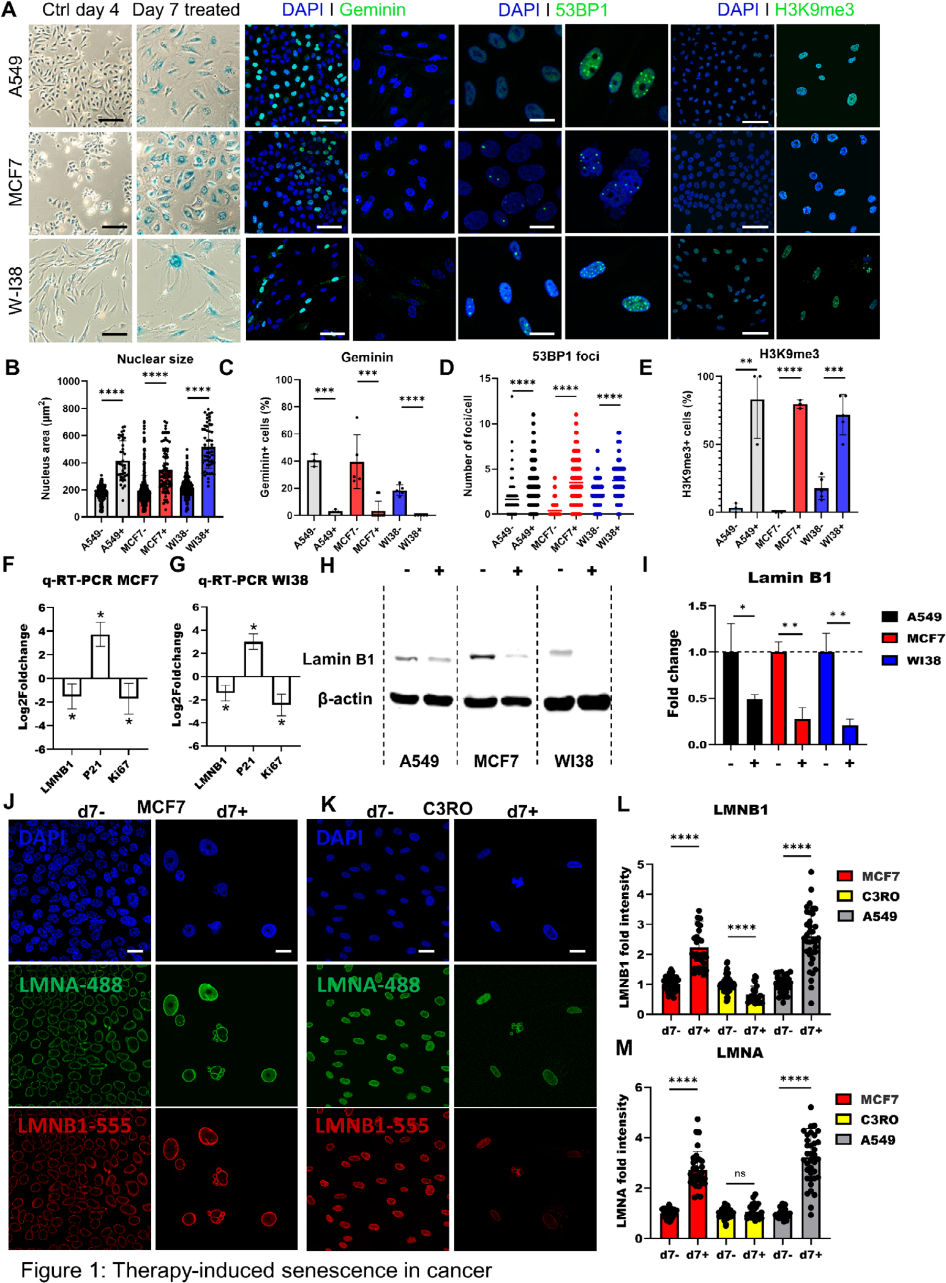
Therapy‐induced senescence in cancer cells and human diploid fibroblasts. A) Representative images of A549, MCF7, and WI‐38 cells, stained for SA‐β‐Gal (left two panels), and overlayed confocal images stained for DAPI (blue) and geminin (green), 53BP1 (green), or H3K9me3 (green) (right panels), 4 days post seeding (non‐treated, left image) and 7 days post treatment (10 Gy, right image). Scale bar: 100 µm for SA‐β‐Gal, geminin and H3K9me3. Scale bar: 25 µm for 53BP1. B) Nuclear size of A549, MCF7 or WI‐38 cells (each dot represents a cell). C) Percentage of geminin+ A549, MCF7 or WI‐38 cells (each dot represents a field of view (FOV)). D) Number of 53BP1 foci per A549, MCF7 or WI‐38 cell (each dot represents a cell). E) Quantification of H3K9me3+ A549, MCF7 or WI‐38 cells (each dot represents a FOV). F) RT‐qPCR analysis of LMNB1, p21, and Ki67 expression in untreated versus 10 Gy‐treated MCF7 cells 7 days post‐treatment, with ACTB as the housekeeping gene (n = 3). G) RT‐qPCR analysis of LMNB1, p21, and Ki67 expression in untreated versus 10 Gy treated WI38, 7 days post‐treatment, with ACTB as housekeeping gene (n = 3). H) Immunoblot of A549, MCF7 and WI‐38 for LMNB1, 7 days post seeding (non‐treated, d7‐) and 7 days post treatment (10 Gy, d7+). I) Immunoblot quantification of A549, MCF7 and WI‐38 for LMNB1, 7 days post seeding (non‐treated, d7‐) and 7 days post treatment (10 Gy, d7+) (n = 3). J) Representative confocal images of MCF7 cells, untreated or 10 Gy‐treated, stained for LMNA (green), LMNB1 (red) and DAPI (blue) on day 7. Scale bar = 25 µm. K) Representative confocal images of C3RO cells, untreated or doxorubicin‐treated (75 nM, continuous), stained for LMNA (green), LMNB1 (red) and DAPI (blue) on day 7. Scale bar = 25 µm. L) Mean intensity of LMNB1 IF signal in MCF7, C3RO and A549 cells, 7 days post seeding (non‐treated, d7‐) and 7 days post treatment (d7+). M) Mean intensity of LMNA IF signal in MCF7, C3RO and A549 cells, 7 days post seeding (non‐treated, d7‐) and 7 days post treatment (d7+). B–E, F, I, and L, M, data was presented with mean and error bars showing standard deviation. Statistics: unpaired parametric two‐tailed Student's *t*‐tests; ns. *p*>0.05; * *p* <0.05; ** *p* <0.01; *** *p* <0.001; **** *p* <0.0001.

We conducted extensive validation experiments to confirm that exposure to 10 Gy of ionizing radiation reliably induces cellular senescence in MCF7 and A549 cells and compared the results to WI‐38. Seven days post‐irradiation, all three cell types exhibited a robust senescent phenotype, as evidenced by multiple well‐established hallmarks of senescence, including increased SA‐β‐Gal staining (**Figure** **1**A), increased nuclear size (Figure 1B), reduced proliferation as indicated by fewer geminin+ cells compared to untreated cells (Figure 1A,C), and an increase in 53BP1 foci (Figure 1A,D). Additionally, we observed enrichment in H3K9me3 (Figure 1A) and a higher percentage of cells with upregulated H3K9me3 staining 7 days after irradiation compared to the proliferating control (Figure 1E), which are established senescence markers.^[^ ^4^ ^]^ Furthermore, 10 Gy‐irradiated MCF7 cells displayed decreased mRNA‐expression of LMNB1 and Ki67 and increased p21 mRNA‐expression, also displayed by senescent WI‐38 fibroblasts (Figure 1F,G).

Loss of lamin B1 protein expression is an acknowledged marker for multiple forms of senescence including damage‐induced, replicative and oncogene‐induced senescence.^[^ ^10^ ^]^ Indeed, we observed lamin B1 loss in irradiated WI‐38 and IMR90 (Figure S1A,B, Supporting Information). We also found lamin B1 loss in irradiated cancer cell models A549 and MCF7 using immunoblotting (Figure 1H,I). In addition, we studied senescence induction in C3RO, a hTERT‐immortalized skin fibroblast, wild‐type p53/p16. Despite being immortalized, C3RO retains key features of primary senescent fibroblasts, as TERT expression does not affect damage‐induced senescence.^[^ ^25^, ^26^ ^]^ Senescence was induced in C3RO using doxorubicin, confirmed by SA‐β‐Gal staining after 7 days (Figure S1C,D, Supporting Information). While LMNB1 protein levels were reduced, both senescent MCF7 and A549 cancer cells displayed increased nuclear staining for both LMNA and LMNB1 (Figure 1J,L,M). This unexpected staining pattern contrasts with that observed in senescent primary fibroblasts, including WI‐38, IMR90, and hTERT‐immortalized C3RO cells, where LMNA and LMNB1 staining intensity decreased, consistent with typical senescence‐associated nuclear lamina remodeling (Figure 1K–M; Figure S1E–G, Supporting Information). These results reveal a previously unrecognized feature of the senescent cancer cells tested here: enhanced lamin B1 and A staining, suggesting distinct nuclear lamina remodeling through local accumulation during senescence in cancer cells compared to human diploid fibroblasts. In contrast, in primary senescent cells, the reduction of LMNB1 staining is well characterized.^[^ ^9^, ^10^, ^27^ ^]^

### 2.2 Development of a Senescence Reporter

#### 2.2.1 Choosing the Most Suitable Senescence Reporter Candidate

**Figure 2:**
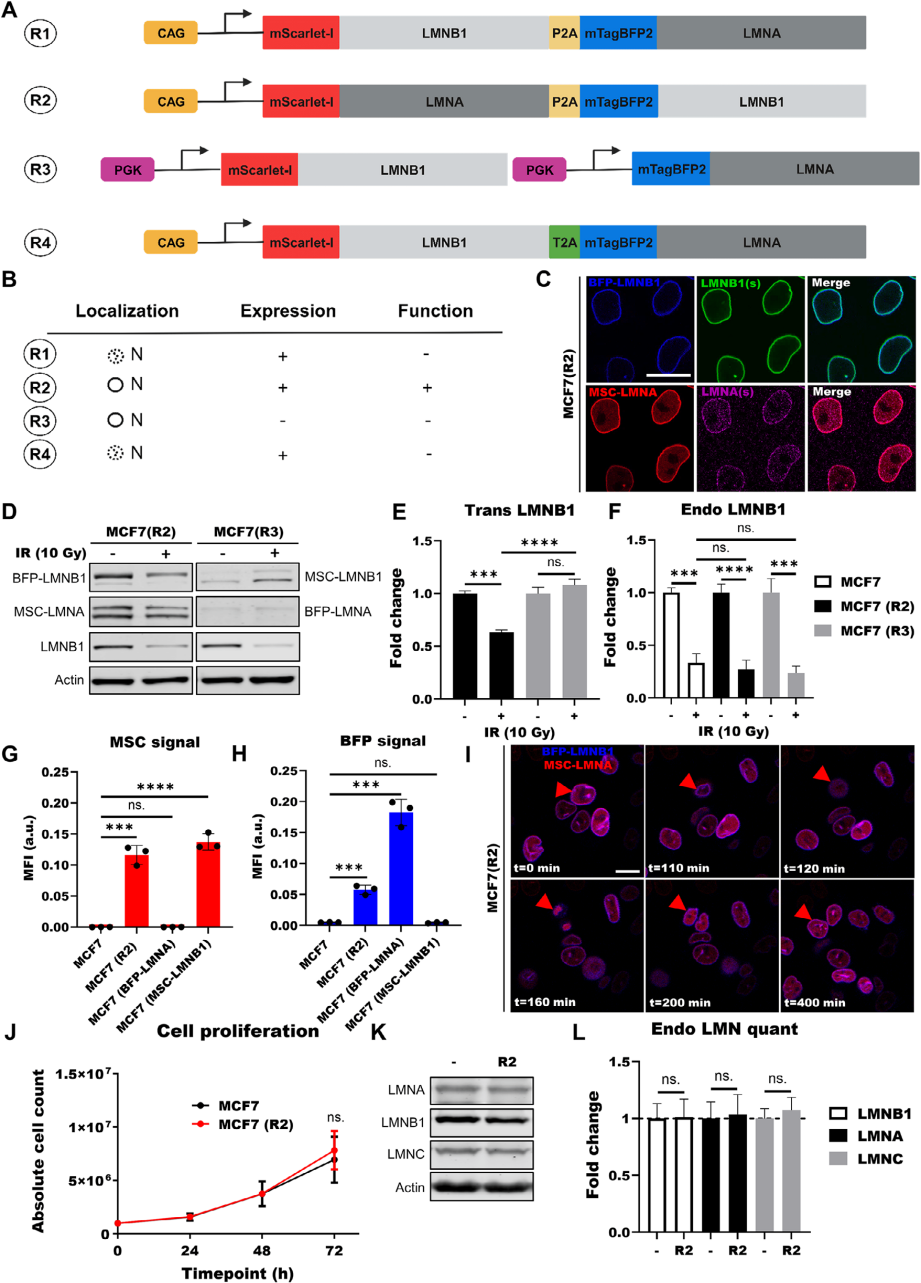
Development of the senescence reporter. A) Reporter candidates R1‐R4 with LMNB1 and LMNA fused to mScarlet‐I (MSC) or mTagBFP2 (BFP). CAG: CAG‐promotor; PGK: PGK‐promotor; P2A: P2A‐linker; T2A: T2A‐linker. B) Reporter criteria, abbreviations are as follows: +, pass; ‐, failure; N, nucleus; O, nuclear lamina; dashes, protein accumulation in nuclear lamina. C) Confocal images of MCF7(LMNB1‐BFP) cells, stained for LMNB1, and MCF7(LMNA‐MSC) cells, stained for LMNA. Scale bar: 25 µm. D) Immunoblot of MCF7(R2) and MCF7(R3) cells for LMNB1‐BFP, LMNA‐MSC, and LMNB1 (endogenous). Loading control: Actin. E) Transgenic LMNB1 protein levels of MCF7(R2) and MCF7(R3) cells 0Gy(‐) and 10 Gy(+), 7 days post‐treatment (n = 3). F) Endogenous LMNB1 protein levels of MCF7, MCF7(R2) and MCF7(R3) cells (n = 3). G) Mean fluorescence intensity (MFI) of MSC in untreated MCF7, MCF7(R2), MCF7(BFP‐LMNA) and MCF7(MSC‐LMNB1) cells measured by flow cytometry. H) MFI of BFP in untreated MCF7, MCF7(R2), MCF7(BFP‐LMNA) and MCF7(MSC‐LMNB1) cells. I) Confocal time‐lapse (0–400 min) of MCF7(R2) cells. J) Growth curve of MCF7 and MCF7(R2) cells, measured by absolute cell numbers at 24, 48, and 72 h after seeding (n = 3). K) Immunoblot of MCF7 and MCF7(R2) cells for endogenous LMNA, LMNB1 and LMNC. Loading control: Actin. L) Endogenous lamin protein‐levels in MCF7 and MCF7(R2) cells (n = 3). E, F, G, H, L, data was presented with mean and error bars showing standard deviation. Statistics: unpaired parametric two‐tailed Student's *t*‐tests; ns. *p* >0.05; *** *p* <0.001; **** *p* <0.0001.

The change in lamin intensities in senescent cancer cells inspired us to develop a senescence reporter with transgenic lamins to identify and sort senescent cells. To this end, we developed reporter candidates for ectopic expression of lamin B1‐ and lamin A fluorescent fusion proteins under a synthetic promoter without the 5′UTR or 3′UTR regions of the endogenous lamin transcripts (**Figure** **2**A). We also included transgenic lamin A in the reporter to not only rely on the lamin B1 signal. We used two cancer models with different p53 status to investigate the behavior of the reporter candidates. We therefore stably integrated the reporter candidates in MCF7 and DU145 (prostate cancer; p53‐ and p16‐mutated) cells using PiggyBac transposase^[^ ^28^ ^]^ and determined the most suitable candidate based on the following criteria: 1) localization, 2) expression and 3) function of transgenic lamins. Reporter candidates R2 and R3 showed good nuclear membrane integration, similar to endogenous lamins, whereas R1 and R4 showed interrupted nuclear membrane staining in blue and red, indicating both transgenic lamins were not properly integrated in the nuclear membrane (Figure 2B,C; Figure S2A–D, Supporting Information). Expression of both transgenic lamins in cells transfected with R3 was much lower compared to R2 and the low expression made R3 unsuitable for confocal imaging (Figure S2C, Supporting Information). Moreover, upon senescence induction of MCF7 cells with 10 Gy (confirmed by the significant loss of endogenous lamin B1 expression) transgenic lamin B1 expression decreased in cells transfected with R2 whereas cells expressing R3 showed no difference in transgenic lamin B1 expression. We quantified the intensity of both upper and lower bands together (Figure 2D–F). Based on the above‐mentioned three criteria (summarized in Figure 2B), we chose candidate R2 as our senescence reporter (SRbr). With flow cytometry we confirmed that fluorescence intensities of mScarlet‐lamin A and BFP‐lamin B1 fusion proteins were suitable for FACS‐sorting (Figure 2G,H).

#### 2.2.2 Transgenic Lamin Behavior in Cancer Cells

As lamins play a role in multiple cellular functions including cell division, DNA repair, chromatin organization and gene‐regulation,^[^ ^29^ ^]^ we wanted to confirm that ectopic expression of lamin fusion proteins did not influence cell behavior. We made cell lines stably‐expressing mScarlet‐lamin B1 or BFP‐lamin A: MCF7^(mSc‐lamin‐B1)^, DU145^(mSc‐lamin‐B1)^, MCF7^(BFP‐lamin‐A)^ and DU145^(BFP‐lamin‐A)^. Both transgenic lamins were expressed 2‐2.5 times higher than the endogenous lamins (Figure S2E,F, Supporting Information). To study transgenic lamin behavior upon senescence, we treated DU145, MCF7, DU145^(mSc‐lamin‐B1)^, MCF7^(mSc‐lamin‐B1)^, DU145^(BFP‐lamin‐A)^ and MCF7^(BFP‐lamin‐A)^ cells for 7 days with 100 nM (DU145) or 25 nM (MCF7) doxorubicin. We confirmed senescence induction based on loss of endogenous lamin B1 protein expression (Figure S2G,H,K,L, Supporting Information, respectively). Further, endogenous lamin B1 loss upon senescence induction was similar for transfected and untransfected DU145 and MCF7 cells, confirming that the incorporation of transgenic lamin did not influence endogenous lamin B1 loss during senescence (Figure S2H,L, Supporting Information). Moreover, transgenic lamin B1 expression was reduced upon senescence induction, although not to the same extent as endogenous lamin B1 (Figure S2I,J,M,N, Supporting Information). Also, incorporation of transgenic lamins did not influence their colony‐forming ability, cell doubling‐rate and proliferative capacity (Figure S2O,R, Supporting Information). To determine whether ectopic lamin expression may influence sensitivity to DNA double‐strand break (DSB)‐inducing agents, we irradiated cells with 2 Gy inducing 53BP1 foci (a marker for DSBs). We performed survival assays using various doses of X‐ray or different concentrations of cisplatin to induce DNA damage. We did not observe biologically relevant differences between transfected and untransfected DU145 and MCF7 cells in the number of DSBs (Figure S2S,T, Supporting Information) and survival curves of transfected DU145 and MCF7 cells did not show altered sensitivity compared to untransfected cells after irradiation and cisplatin treatment (Figure S2U–X, Supporting Information). Both transgenic lamins showed proper degradation of and incorporation in the nuclear envelop during the cell cycle of MCF7(SRbr) cells (Figure 2I). Furthermore, integration of transgenic lamins did not influence endogenous lamin B1 protein loss in these cells (Figure 2F), their cell doubling‐rate (Figure 2J) or expression of endogenous lamin B1, ‐A and ‐C (Figure 2K,L). Altogether, these results indicate that incorporation of transgenic lamins in MCF7 and DU145 cells does not influence their behavior for the parameters investigated.

### 2.3 Analysis of Senescence in Cancer Cells

#### 2.3.1 Screening for Senescence in Cancer

**Figure 3:**
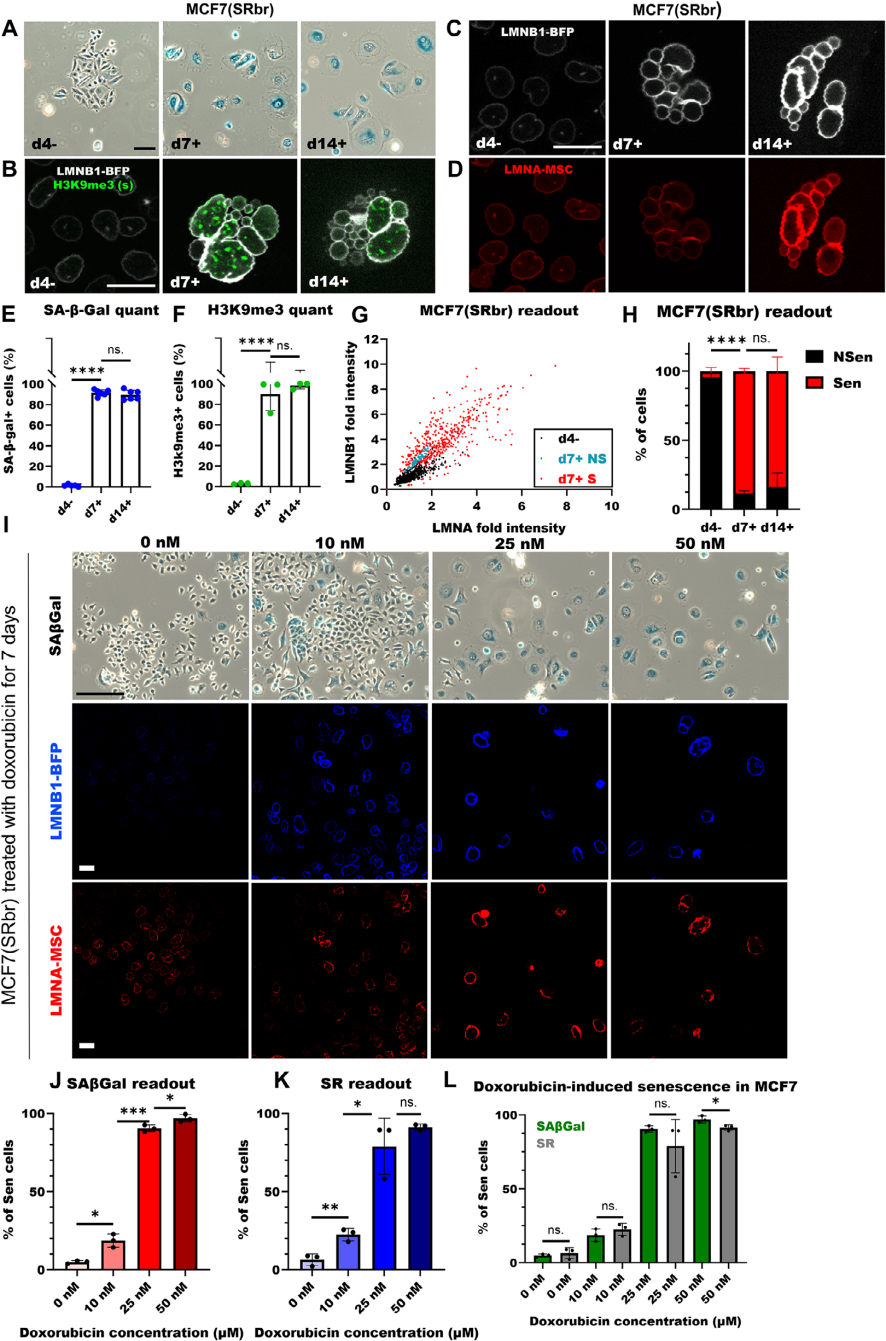
Analysis of senescence in cancer cells. A) Images of untreated (d4−) and 10 Gy‐treated MCF7(SRbr) cells 7 and 14 days post‐treatment (d7+; d14+), stained for SA‐β‐Gal. Scale bar: 100 µm. B) Confocal images of untreated (d4−) and 10 Gy‐treated (d7+ and d14+) MCF7(SRbr) cells, displaying H3K9me3 staining and LMNB1‐BFP. Scale bar: 25 µm. C) Untreated and 10 Gy‐treated MCF7(SRbr) cells, displaying LMNB1‐BFP. Scale bar: 25 µm. D) Untreated and 10 Gy‐treated MCF7(SRbr) cells, displaying LMNA‐mScarlet (MSC). Scale bar: 25 µm. E) Percentage of SA‐β‐Gal+ MCF7(SRbr) cells (each dot represents one FOV). F) Percentage of H3K9me3+ MCF7(SRbr) cells (each dot represents a FOV). G) Scatterplot of MCF7(SRbr) cells, 4 days post seeding (non‐treated, d4−) and 7 days post treatment (10 Gy, d7+). H) Quantification of senescent and non‐senescent MCF7(SRbr) cells in untreated and 10 Gy‐treated cells determined with screening assay. I) Images of untreated (0 nM) and doxorubicin‐treated MCF7(SRbr) cells 7 days post‐treatment (10, 25, or 50 nM), stained for SA‐β‐Gal (upper panel, scale bar: 100 µm), confocal images displaying LMNB1‐BFP (middle panel, scale bar: 25 µm), and LMNA‐MSC (bottom panel, scale bar: 25 µm). J) Percentage of SA‐β‐Gal+ MCF7(SRbr) cells (each dot represents one FOV). K) Quantification of senescent and non‐senescent MCF7(SRbr) cells in untreated and doxorubicin‐treated cells determined with screening assay. L) Comparison of the percentage of SA‐β‐Gal+ (green) and senescent MCF7(SRbr) cells identified by the screening assay (gray). E, F, H, and J–L, Data was presented with mean and error bars showing standard deviation. Statistics: unpaired parametric two‐tailed Student's *t*‐tests; ns. *p*>0.05; * *p* <0.05; ** *p* <0.01; *** *p* <0.001; **** *p* <0.0001.

Therapy‐induced senescence is a promising approach to treat cancer, but has also been linked to tumor relapse, aggressiveness and metastasis,^[^ ^12^, ^18^ ^]^ emphasizing the importance of monitoring senescence in cancer. Here, we used our senescence screening method to identify senescent cancer cells. To induce senescence, we irradiated MCF7(SRbr) cells with 10 Gy and found more SA‐β‐Gal+ cells (**Figure** **3**A,E), SAHF‐formation, characterized by enrichment in H3K9me3 (Figure 3B), and a higher percentage of cells with upregulated H3K9me3 staining (Figure 3F) 7 and 14 days after irradiation compared to the proliferating control (d4‐). In 10 Gy‐irradiated MCF7(SRbr) cells, transgenic lamin A and lamin B1 intensities profoundly increased at d7 and d14 compared to the proliferating control (Figure 3C,D) similar to what was observed for endogenous lamins in senescent untransfected MCF7 cells (Figure 1J,L,M). Further, we observed a linear correlation between lamin A and lamin B1 intensities for proliferating cells (Figure 3G). Based on those observations, we developed an algorithm to distinguish between non‐senescent and senescent cells.

We determined the range of transgenic lamin intensities in proliferating cells and defined the outliers as high lamin intensities (> the mean +2*standard deviation). We categorized non‐senescent cells as cells with lamin intensities within the normal range which follow a linear correlation between lamin B1 and A intensities. Conversely, senescent cells were defined as those with high transgenic lamin intensities or those deviating from the linear correlation (Figure 3G). For each experiment, the threshold for the lamin intensities was determined using the corresponding proliferating control. We found that 7 days after irradiation, over 90% of MCF7(SRbr) were senescent. This percentage was not significantly increased on day 14 after irradiation (Figure 3H). Similarly, 10 Gy‐irradiated A549(SRbr) and DU145(SRbr) cells displayed profoundly higher lamin intensities than untreated cells (Figure S3A,B,D,E, Supporting Information respectively), and our senescence screening assay detected that most cells were senescent at d7 and d14 (Figure S3C,F, Supporting Information respectively).

Next, we treated MCF7(SRbr) cells with various concentrations of doxorubicin. To estimate the accuracy of our assay, we directly compared the SRbr readouts with SA‐β‐Gal staining results from the same sample and observed an increase in the number of SA‐β‐Gal+ cells and cells with higher lamin intensities after exposure to increasing doses of doxorubicin (Figure 3I,J). Moreover, we found that our assay readout was very comparable to the SA‐β‐Gal staining results (Figure 3J–L). Only at the highest doxorubicin dose, the senescence assay underestimated the percentage of senescent cells compared to the SA‐β‐Gal staining results (Figure 3L).

Transgenic lamins are constitutively expressed by the cells, as our senescence reporter construct contains a CAG promoter for the ectopic expression of transfected lamin B1 and A fluorescent fusion proteins without the 5′UTR or 3′UTR of the lamins. Despite this, we have shown that the upregulation of transgenic lamin intensities is associated with senescence in cancer cells. With our senescence screening assay, we are able to quantify the number of senescent cells in a dose‐dependent manner.

#### 2.3.2 PiggyBac versus Safe Harbor Integration of the Senescence Reporter in Cancer Cells

To exclude that the effect of changes in transgenic lamin expression in senescence are due to the copy number or location of plasmid integration in the genome, we used CRISPR/Cas9 to integrate a single copy of the SRbr sequence in a safe harbor locus (AAVS1) of the genome (MCF7(SRsh)). This locus is regarded as a genomic safe harbor because integration at this site does not interfere with endogenous gene expression, as shown in various cell lines, including MCF7.^[^ ^30^, ^31^ ^]^

In the MCF7(SRbr) cell line, the reporter plasmid was integrated using the PiggyBac transposon system, which results in semi‐random genomic integration. Although the number of integrated copies is expected to be low due to the plasmid‐to‐transposase ratio used, it cannot be precisely controlled. In contrast, the safe harbor approach ensures the presence of only one or two copies of the SRbr plasmid per cell, depending on whether the cell is heterozygous or homozygous for the insertion.

We irradiated MCF7(SRsh) cells with 10 Gy and found that transgenic lamin intensities increased similarly to those in 10‐Gy‐irradiated MCF7(SRbr) cells (Figure S3G,H, Supporting Information) and that 90% of cells were senescent (Figure S3I, Supporting Information). This confirms that the locus of integration of the SR plasmid DNA does not influence senescence‐associated upregulation of the transgenic lamin intensities, validating the robustness of our system.

### 2.4 IL6‐Upregulation in Senescent Cancer Cells

The SASP is associated with and present in specific types of senescence.^[^ ^32^ ^]^ SASP may influence neighboring cells by promoting epithelial cell proliferation, cell migration and invasion and can alter the differentiation status of neighboring cells.^[^ ^33^ ^]^ Although SASP consists of many components, IL6 is its most prominent cytokine, which has been associated with damage‐induced senescence in various types of primary cells.^[^ ^33^ ^]^ To study SASP‐regulation in senescent cancer cells, we used IL6‐upregulation in senescent cells as an indication of SASP‐activation.^[^ ^33^, ^34^ ^]^ To this end, we extended the SRbr reporter with nuclear GFP expressed under a human IL6 promoter (named SRs; Figure S4A, Supporting Information). First, we tested IL6 promoter‐response by treating DU145^(IL6REP)^ cells (Figure S4B, Supporting Information) with lipopolysaccharide (LPS) to induce IL6‐expression.^[^ ^35^, ^36^ ^]^ Upon LPS treatment, we found increased GFP intensity and percentage of GFP+ cells (Figure S4C–E, Supporting Information), corresponding to increased IL6 and GFP mRNA‐expression (Figure S4F, Supporting Information), suggesting the introduced promoter responded similarly to the endogenous IL6 promoter. Upon 7‐day doxorubicin treatment of DU145^(IL6REP)^, we observed increased GFP intensity in a fraction of the cells (Figure S4G,H, Supporting Information), suggesting upregulated IL6‐expression in those cells.

**Figure 4:**
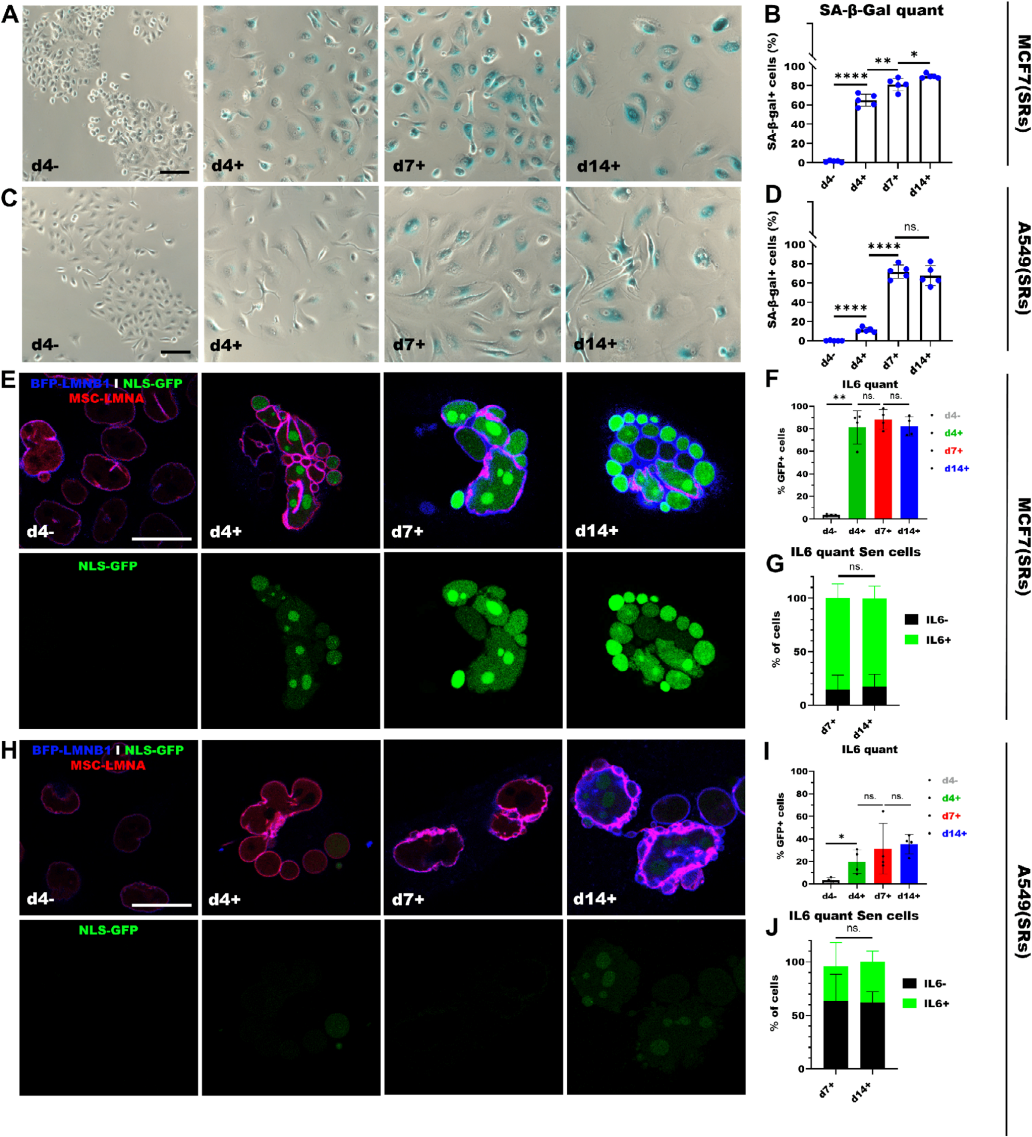
IL6‐upregulation in senescent cancer cells. A) Images of untreated (d4‐) and 10 Gy‐treated MCF7(SRs) cells 4, 7 and 14 days post‐treatment (d4+; d7+; d14+), stained for SA‐β‐Gal. Scale bar: 100 µm. B) Percentage of SA‐β‐Gal+ MCF7(SRs) cells (each dot represents one FOV). C) Images of A549(SRs) cells stained for SA‐β‐Gal. D) Percentage of SA‐β‐Gal+ A549(SRs) cells (each dot represents one FOV). E) Confocal images of untreated and 10 Gy‐treated MCF7(SRs) cells at d4+, d7+, and d14+. Scale bar: 25 µm. F) Percentage of IL6+ (NLS‐GFP) MCF7(SRs) cells. G) Percentage of IL6± MCF7(SRs) cells within the senescent population (n = 3). H) Confocal images of untreated and 10 Gy‐treated A549(SRs) cells. I. Percentage of IL6+ (NLS‐GFP) A549(SRs) cells. J. Percentage of IL6± A549(SRs) cells within the senescent population (n = 3). B, D, F, G, I and J, data was presented with mean and error bars showing standard deviation. Statistics: unpaired parametric two‐tailed Student's *t*‐tests; ns. *p*>0.05; * *p* <0.05; ** *p* <0.01; **** *p* <0.0001.

To determine the SASP‐profile of senescent cancer cells, we exposed MCF7(SRs) and A549(SRs) to 10 Gy irradiation. Both the SA‐β‐Gal staining and our senescence screening assay showed that almost all MCF7(SRs) and A549(SRs) cells were senescent at d7 and d14 (**Figure** **4**A–D; Figure S4I,J, Supporting Information respectively). Four days after irradiation, we already found an increase in SA‐β‐Gal+ MCF7(SRs) and A549(SRs) cells, although cells appeared to be in a premature state of senescence with less profound staining (Figure 4A–D). These pre‐senescent MCF7(SRs) displayed IL6‐upregulation compared to the control with no further increase in GFP+ cells over time (Figure 4E,F). In pre‐senescent A549(SRs), we observed a similar trend, but the percentage of GFP+ cells was much lower compared to MCF7(SRs) (Figure 4H,I). Within the senescent MCF7(SRs) population we found that most senescent cells on day 7 and day 14 displayed IL6‐upregulation (IL6+) compared to the control (Figure 4G). On the other hand, the majority of senescent A549(SRs) cells did not upregulate IL6 (IL6‐; Figure 4J). As SASP has been linked to tumor progression and may have detrimental effects on the tumor microenvironment, understanding the SASP‐profile of cancers will give more insight into the possible effects of senescence in these tumor types and whether TIS may be a sensible treatment option. SRs could be a valuable tool to study these differences in SASP‐profiles.

### 2.5 Sorting Senescent and Non‐Senescent Cancer Cells

**Figure 5:**
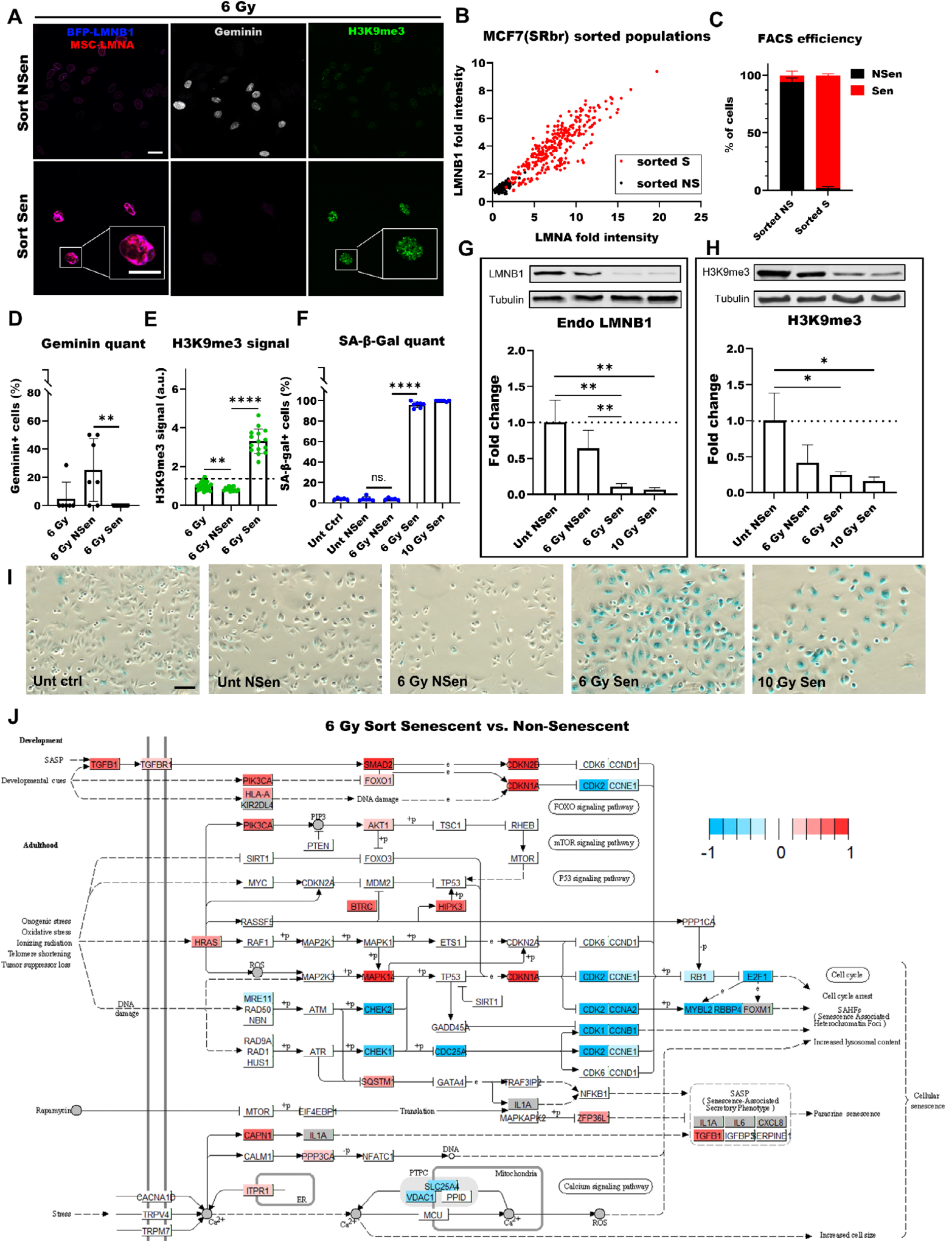
Sorting senescent versus non‐senescent cells. A) Confocal images of 6 Gy‐treated, sorted MCF7(SRbr) cells, categorized as non‐senescent (Sort NSen) versus senescent (Sort Sen) based on LMNA‐MSC‐ and LMNB1‐BFP intensities 8 days post‐treatment co‐stained for geminin and H3K9me3. Scale bar: 25 µm. B) Scatterplot of LMNA‐MSC and LMNB1‐BFP intensities from confocal images of sorted MCF7(SRbr) cells (each dot represents a cell). C) Quantification of senescent and non‐senescent cells in Sort NSen and Sort Sen populations determined with screening assay (n = 3). D) Percentage of geminin+ cells of 6 Gy‐treated, sorted MCF7(SRbr) cells (each dot represents a FOV). E) Quantification of H3K9me3 signal intensity in 6 Gy‐treated, sorted MCF7(SRbr) cells (each dot represents a cell). F) Percentage of SA‐β‐Gal+ MCF7(SRbr) cells 8 days post‐treatment (each dot represents a FOV). G) Representative immunoblot and quantification for endogenous LMNB1 in sorted MCF7(SRbr) cells (n = 3). Loading control: Tubulin. Protein bands are not in original order. H) Representative immunoblot and quantification for H3K9me3 in sorted MCF7(SRbr) cells (n = 3). Loading control: Tubulin. Protein bands are not in original order. I) Images of sorted MCF7(SRbr) cells 8 days post‐treatment (6 or 10 Gy), stained for SA‐β‐Gal. Scale bar: 100 µm. J) KEGG pathway of Cellular Senescence (0 4218). Genes are colored based on RNA‐sequencing results of 6 Gy‐treated, sorted and categorized as senescent versus non‐senescent populations. Gray genes are not sufficiently detected in the data in both sample groups. Unt = untreated, Ctrl = not sorted, Sort NSen = sorted, categorized as non‐senescent, Sort Sen = sorted, categorized as senescent based on transgenic LMNA and LMNB1 intensity. C–H, data was presented with mean and error bars showing standard deviation. Statistics: unpaired parametric two‐tailed Student's t‐tests; ns. *p*>0.05; * *p* <0.05; ** *p* <0.01; **** *p* <0.0001.

To further characterize senescent cancer cells, we developed a method to sort senescent cells and non‐senescent cells using FACS. First, we validated that the intensities (measured by flow cytometry) of mScarlet‐lamin A and BFP‐lamin B1 in 10 Gy‐irradiated cells increased significantly compared to untreated cells (Figure S5A,B, Supporting Information). When exposing cells to 6 Gy, we obtained a mix of non‐senescent and senescent cells, indicated by fewer SA‐β‐Gal+ cells compared to 10 Gy‐irradiated cells (Figure S5C,G, Supporting Information). Next, we exposed MCF7(SRbr) to 6 or 10 Gy and sorted cells 7 days later based on their BFP‐lamin B1 and mScarlet‐lamin A intensities. With the applied settings, we did not find senescent cells in the untreated population. On average, we obtained populations with 7% and 12% of cells sorted as senescent and 22% and 12% sorted as non‐senescent for 6 and 10 Gy, respectively. The sorted cells were replated, then fixed and stained for senescence markers the next day. As expected, we observed low and high lamin intensities for the non‐senescent and senescent cells, respectively (**Figure** **5**A,B; Figure S5D, Supporting Information). Our senescence screening assay accurately verified the sorted populations: the non‐senescent cells were confirmed as non‐senescent, and the senescent cells as senescent (Figure 5C; accuracy of 96%).

We found geminin+ cells in the sorted non‐senescent population, indicative of cell proliferation, whereas the senescent population of 6 and 10 Gy did not contain geminin+ cells (Figure 5A,D; Figure S5D,E, Supporting Information). We also observed SAHFs in the senescent population, while absent in the non‐senescent population (Figure 5A,E; Figure S5D,F, Supporting Information). Moreover, considerably more SA‐β‐Gal+ cells were present in the 6 and 10 Gy senescent populations compared to the 6 Gy non‐senescent population, which was similar to the untreated non‐senescent population (Figure 5F,I). Immunoblotting showed a decrease in lamin B1 and H3K9me3 expression in 6 and 10 Gy‐sorted senescent cells compared to untreated non‐senescent cells (Figure 5G,H). Importantly, expression of lamin B1 was lower in 6 Gy‐treated senescent cells compared to 6 Gy‐treated non‐senescent cells (Figure 5G).

Next, we performed mRNA sequencing on sorted 6 Gy‐treated non‐senescent cells and 6 Gy‐treated senescent cells from three independent experiments. Differential gene‐expression analysis showed that 40 out of 91 detected senescence‐associated genes, defined by the KEGG pathway Cellular Senescence (0 4218), were differentially expressed when comparing non‐senescent and senescent cells, with 19 genes downregulated and 21 upregulated (Figure 5J). Moreover, senescent cells displayed reduced expression of genes specific to G2/M‐ and S‐phase (Figure S5H, Supporting Information), suggesting that most senescent cells were arrested in G1‐phase. Pathway analysis further revealed the downregulation of several cell‐cycle‐related pathways and upregulation of pathways related to cell adhesion and wound healing in 6 Gy‐treated senescent cells compared to 6 Gy‐treated non‐senescent cells (Figure S5I, Supporting Information). Altogether, our results indicate that our sorting protocol is a good method to collect non‐senescent and senescent cells for further analysis.

### 2.6 Monitoring Senescence in Cancer Cells

#### 2.6.1 Senescence Induction in Cancer Cells Using Doxorubicin

**Figure 6:**
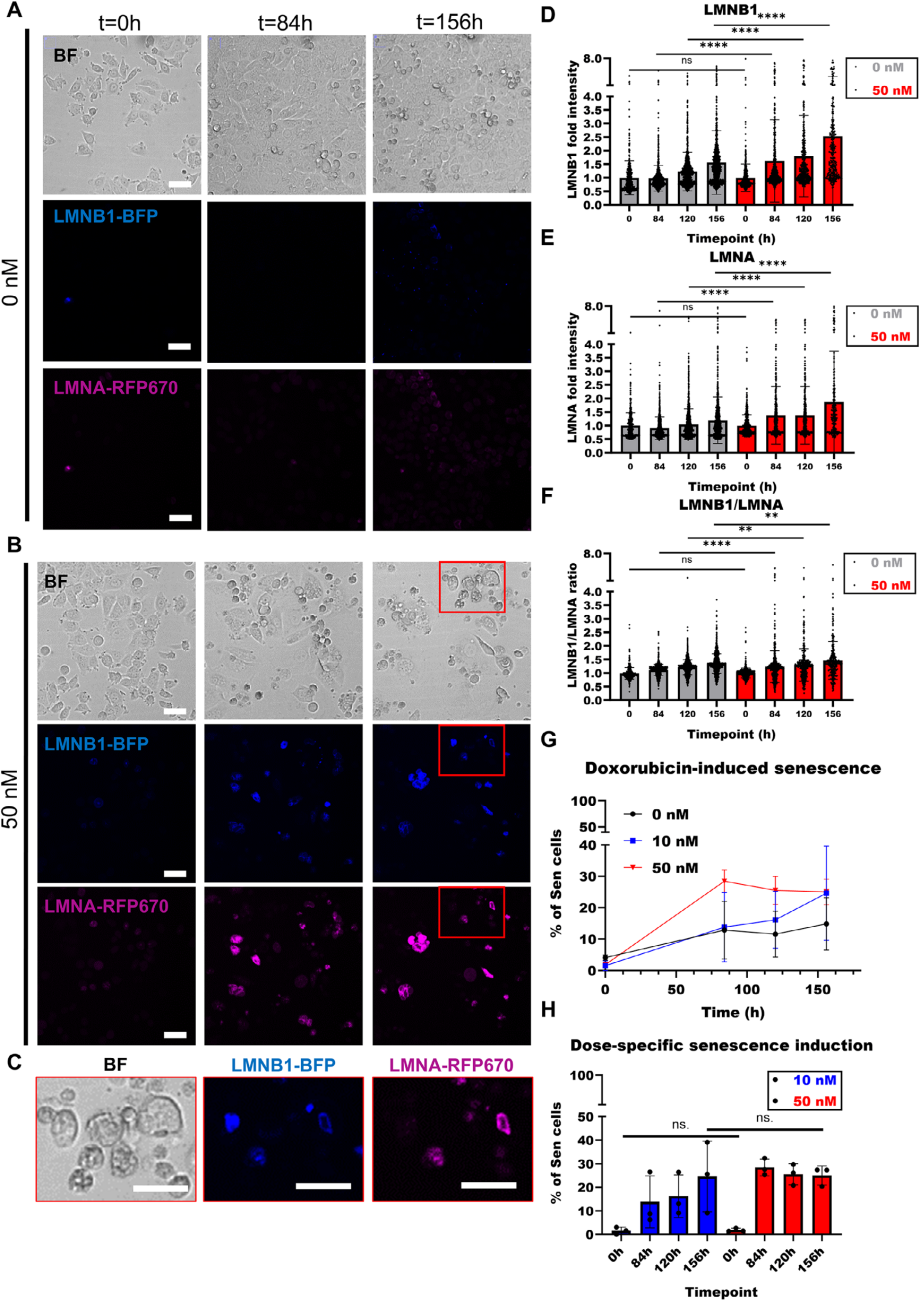
Monitoring senescence induction. A) Time‐lapse images of untreated (0 nm) MCF7(SRbfr) cells, imaged in the bright‐field channel (BF), LMNB1‐BFP (blue), and LMNA‐RFP670 (pink) channel. Three time points are shown: 0, 84, and 156 h. Scale bar = 50 µm. B) As in A, for doxorubicin‐treated (50 nm) MCF7(SRbfr) cells. C) Zoom‐in of the last timepoint (t = 156 h) 50 nm‐treated MCF(SRbfr) cells, from the red rectangle in Figure A. Scale bar = 50 µm. D. LMNB1 intensities over time for untreated (0 nm) and doxorubicin‐treated (50 nm) MCF7(SRbfr) cells (t = 0 h, t = 84 h, t = 120 h, and t = 156 h). Each dot represents a single cell. E) LMNA intensities over time for untreated (0 nM) and doxorubicin‐treated (50 nM) MCF7(SRbfr) cells (t = 0 h, t = 84 h, t = 120 h, and t = 156 h). Each dot represents a single cell. F. Ratio of LMNB1/LMNA intensities over time for untreated (0 nM) and doxorubicin‐treated (50 nM) MCF7(SRbfr) cells (t = 0 h, t = 84 h, t = 120 h, and t = 156 h). Each dot represents a single cell. G. Percentage of senescent cells over time (t = 0 h, t = 84 h, t = 120 h, and t = 156 h), as determined by the screening assay in two (0 nM) or three (10, 50 nM) independent experiments. H) Percentage of senescent cells (10, 50 nm) over time (t = 0 h, t = 84 h, t = 120 h, and t = 156 h). Each dot represents one independent experiment. D–F and H, data was presented with mean and error bars showing standard deviation. Statistics: unpaired parametric two‐tailed Student's t‐tests; ns. *p* >0.05; ** *p* <0.01; **** *p* <0.0001.

To investigate the dynamic behavior of the lamins during senescence induction, we used a new version (SRbfr, blue‐far red) of our fluorescent reporter construct in MCF7 cells. SRbfr contains LMNB1 fused to BFP, LMNA fused to RFP670 (replacing mScarlet to reduce spectral bleed‐through), and mScarlet fused to an NLS to mark nuclei for image analysis. To assess senescence induction, we monitored MCF7(SRbfr) cells for seven days following treatment with varying concentrations of doxorubicin (0, 10, and 50 nm). Upon doxorubicin exposure, we observed a progressive increase in the fluorescence intensity of the transgenic lamins LMNB1‐BFP and LMNA‐RFP670 (**Figure** **6**A–E; Figure S6, Supporting Information). Significant increases in LMNB1 and LMNA intensities compared to untreated controls were first detected at 84 h post‐treatment and persisted at subsequent time points (Figure 6D,E). In addition, the LMNB1/LMNA intensity ratio was significantly elevated in 50 nm‐treated cells relative to untreated cells from 84 h onwards (Figure 6F), suggesting a change in the correlation between lamin B1 and lamin A intensities, the basis of our senescence screening assay.

We quantified the fraction of senescent cells in each condition using our senescence screening assay. This fraction gradually increased in cells treated with 10 nm doxorubicin, whereas it grew rapidly in the 50 nm condition before plateauing (Figure 6G,H). Notably, the percentage of senescent cells in the 50 nm‐treated population at 156 h was lower than we previously observed on day 7 (Figure 3K,L). The most plausible explanation for this observation is that a subset of cells underwent apoptosis rather than entered senescence, likely as a consequence of the relatively high dose. In the previous experiment (Figure 3K,L) the cells were fixed and apoptotic cells have been washed away, resulting in a high percentage of senescent cells. A modest increase in the fraction of senescent cells was also observed in untreated controls, most likely due to the cultures reaching over‐confluence. However, this increase plateaued after 84 h and remained stable at a slightly elevated level thereafter (Figure 6G). These findings demonstrate that our reporter system is able to reliably monitor senescence induction in response to doxorubicin in a dose‐ and time‐dependent manner.

#### 2.6.2 Senescence Escape in Cancer

**Figure 7:**
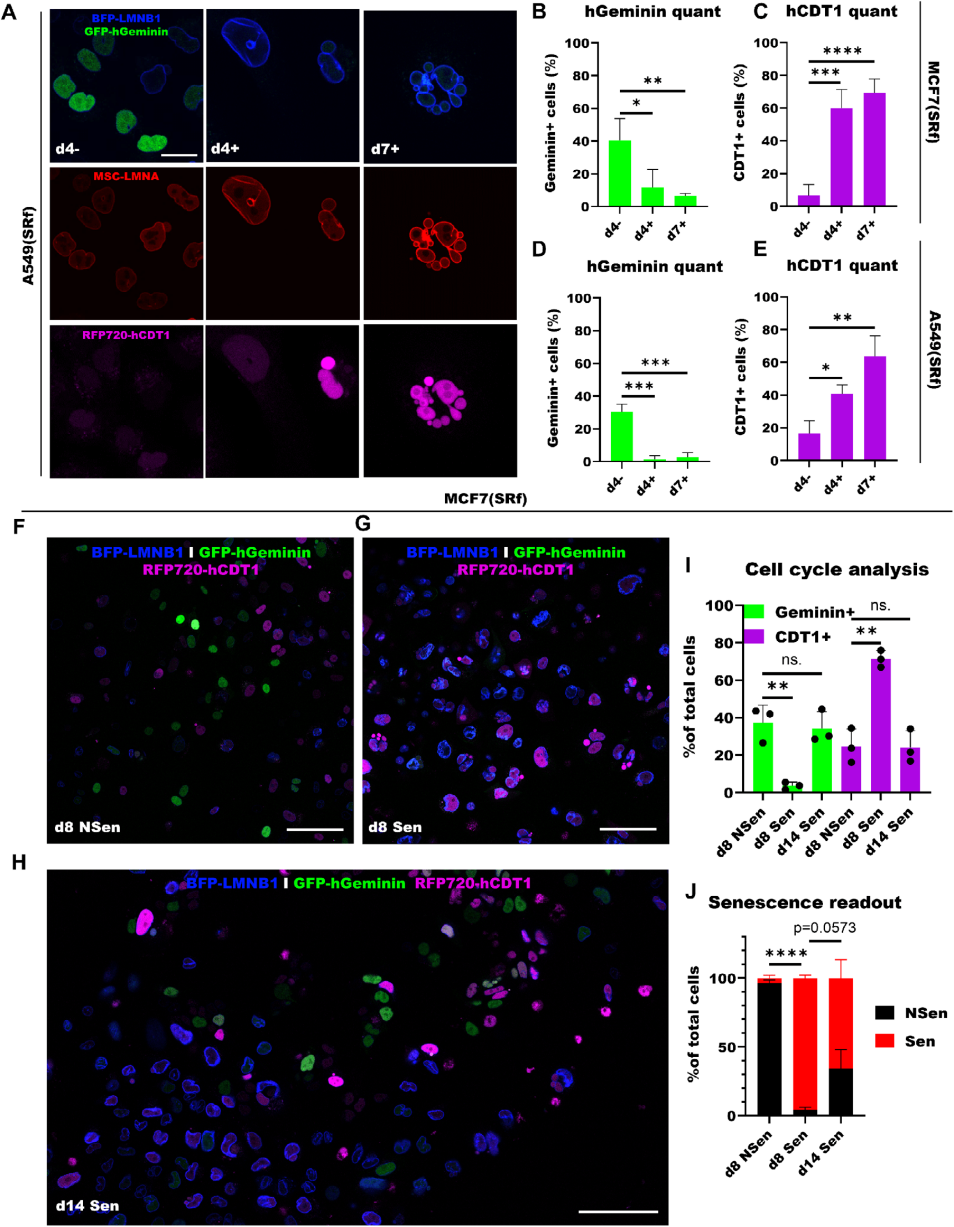
Monitoring senescence induction and escape. A) Confocal images of untreated and 10 Gy‐treated A549(SRf) cells 4 and 7 days post‐treatment. Scale bar: 25 µm. B) Percentage of hGeminin+ cells of untreated and 10 Gy‐treated MCF7(SRf) cells. C) Percentage of hCDT1+ MCF7(SRf) cells. D) Percentage of hGeminin+ cells of untreated and 10 Gy‐treated A549(SRf) cells. E) Percentage of hCDT1+ A549(SRf) cells. F) Confocal image of treated Non‐senescent sorted MCF7(SRf) cells 8 days post‐treatment and one day post‐FACS. Scale bar: 100 µm. G) Confocal image of treated Sort Senescent MCF7(SRf) cells. H) Confocal image of treated Sort Sen cells 14 days post‐treatment and 7 days post‐FACS. I) Percentage of hCDT1± and hGeminin± sorted cells. J) Quantification of senescent and non‐senescent cells in Sort NSen/Sen populations with screening assay (n = 3). B–E, I, and J, data was presented with mean and error bars showing standard deviation. Statistics: unpaired parametric two‐tailed Student's *t*‐tests; ns. *p* >0.05; * *p* <0.05; ** *p* <0.01; *** *p* <0.001; **** *p* <0.0001.

Inducing senescence is a promising anti‐cancer strategy, especially in combination with the use of senolytics.^[^ ^11^, ^12^ ^]^ However, TIS‐escape may lead to unwanted side‐effects such as senescence‐associated reprogramming of cancer cells to a more stem‐like phenotype,^[^ ^37^ ^]^ which may contribute to tumor regrowth and evolution.^[^ ^18^, ^38^ ^]^ To monitor TIS escape in live cells, we created cancer cell models stably‐expressing both SRbr and cell cycle markers (Fucci: the G1 marker iRFP720‐hCDT1(30/120) and the S/G2/M marker GFP‐hGeminin(1/110)): MCF7(SRf) and A549(SRf) (Figure S7A, Supporting Information). We first validated that the transgenic lamins and cell cycle proteins were functional in these cells by performing a time‐lapse experiment with MCF7(SRf), and observed that when cells cycled through the cell cycle, lamins were degraded and re‐integrated into the nuclear membrane and cells lost and regained geminin and CDT1 (Figure S7B, Supporting Information). Next, we investigated the expression of cell cycle markers upon senescence induction with 10 Gy irradiation in A549(SRf) and MCF7(SRf) cells. We found a decrease in geminin+ cells and an increase in CDT1+ cells after irradiation, suggesting that in senescence (d7+) most cells are in G1‐phase of the cell cycle (**Figure** **7**A–E). Next, we monitored senescence in MCF7(SRf) cells. We treated cells with 6 Gy and sorted for senescence after 7 days. After replating the cells, live‐cell imaging at d8 and d14 after irradiation (Figure 7F,G,I) revealed fewer geminin+ and more CDT1+ cells in the senescent population compared to the non‐senescent population at d8. However, at d14 we observed an increase in geminin+ cells, a decrease in CDT1+ cells and an increase in the percentage of non‐senescent cells within the sorted senescent population (Figure 7H–J), suggesting that senescent cancer cells may have escaped their senescent state and re‐entered the cell cycle. Together, these results show that we can monitor TIS using our platform.

## 3 Discussion

Cellular senescence was originally defined by Hayflick in the 1960s as a characteristic of cultured cells that exceeded their replicative limit.^[^ ^23^ ^]^ Nowadays, the definition of senescence has been extended by a state of durable cell cycle arrest inducible by various triggers including replicative stress, oncogene activation, loss of tumor suppressor genes, development, epigenetic influences, mitochondrial dysfunction and DNA damage caused for instance by chemotherapeutic drugs or ionizing radiation.^[^ ^39^ ^]^ These diverse triggers result in distinct molecular and cellular features, contributing to the complexity and heterogeneity of senescence.

A major challenge in the field is the lack of specific, universal biomarkers, which hampers our ability to define and target senescence effectively. To address this, the International Cell Senescence Association proposed a multi‐marker approach with a three‐step workflow: 1) screening for senescence using SA‐β‐Gal and/or lipofuscin, 2) verifying the senescent state with additional nuclear markers (e.g., downregulation of proliferation markers, lamin B1 downregulation) and 3) using markers for specific types of senescence including SASP and DDR.^[^ ^32^ ^]^ Classical staining of SA‐β‐Gal^[^ ^3^, ^40^ ^]^ or lipofuscin,^[^ ^41^, ^42^ ^]^ is not compatible with live‐cell analysis. Consequently, real‐time senescence reporters have been developed,^[^ ^43^ ^]^ including SA‐β‐Gal reporters,^[^ ^44^, ^45^, ^46^ ^]^ and lipofuscin‐binding probes (GLF16 and GL9 as part of the platform GL392).^[^ ^47^, ^48^ ^]^ These lipofuscin‐binding probes are able to detect and isolate senescent cells and may be used for tissues and animal applications. The unique advantage of our developed senescence reporter platform is that it allows live‐cell detection and monitoring of senescence, particularly tracking the senescence induction and escape over time, which is not possible with established probes.

We identified a previously unrecognized feature of senescent cancer cells: an increase in LMNA and LMNB1 nuclear staining intensities despite reduced LMNB1 protein levels by immunoblot, which is in contrast to the typical reduction seen in primary senescent fibroblasts. This seemingly contradictory observation suggests altered nuclear lamin dynamics in senescent cancer cells, possibly due to lamin redistribution by local accumulation. We used this observation as inspiration to develop a lamin‐based reporter for senescence.

Here, we presented a reporter platform that enables reliable detection and sorting of senescent cells in live populations. As our reporter platform detects senescence based on deviations from the typical linear correlation between fluorescent lamin B1 and lamin A intensities observed in proliferating cells, it eliminates the need for a fixed intensity threshold, while still enabling reliable detection of senescent cells relative to the untreated population. Unlike reporters relying on p16, which is often inactivated in cancers, our system functions independently of p16 transcriptional status. Moreover, in transfected cells lamin intensities are always present in contrast to for instance p16 reporters which, although reliably monitor senescent cells in mice,^[^ ^49^, ^50^ ^]^ suffer from poor performance in many cancers due to mutated or absent p16‐expression.^[^ ^51^, ^52^ ^]^ Our reporter system is compatible with both imaging live and fixed cells, and we developed variants with different fluorophore combinations to suit specific applications, including FACS. We simultaneously analyzed senescence‐associated features such as cell cycle status or SASP, allowing us to monitor IL6 expression over time. In contrast, methods like SASP‐RAP rely on chemo‐luminescent assays that require cell fixation and are therefore unsuitable for dynamically assessing SASP activation in single cells.^[^ ^53^ ^]^ By integrating SASP markers and enabling single‐cell analysis, we uncovered heterogeneity both within and between senescent populations, offering new insights into the complexity and implications of TIS.

Our platform also supports early detection of senescence following DNA‐damaging treatment. In MCF7(SRbfr) cells, we observed dose‐ and time‐dependent increases in reporter activity after doxorubicin exposure, with detectable changes emerging as early as 84 h. This sensitivity enables the detection of pre‐senescent or transitional states before full senescence has been established.

Although introducing transgenic lamins via plasmid DNA is straightforward in cancer cell lines, application in primary or patient‐derived cells may require alternative delivery methods, such as lentiviral or RNA transduction. Further, lamin B1 and ‐A are not typically mutated in cancers, but sometimes differentially expressed, highlighting the relevance of controlled expression similar to endogenous levels.^[^ ^54^, ^55^, ^56^ ^]^ While ectopic expression may not fully reflect endogenous lamin levels, our data indicate no interference with cell behavior or treatment sensitivity, making the system robust and informative.

Despite the promising insights gained, it is important to acknowledge a limitation of our study: experiments were confined to a limited number of cancer cell lines. Given the extensive heterogeneity inherent to cancer, it remains unknown to what extent these findings are broadly representative of cancer cell lines in general. Future research must encompass a wider array of cancer models to assess the generalizability of these observations and the utility of our reporter system across diverse cancer contexts.

In conclusion, our reporter is a powerful tool for studying TIS as an anti‐cancer strategy, facilitating evaluation of its therapeutic benefits against the risk of tumor recurrence. It enables live‐cell monitoring, high‐content screening, single‐cell resolution, and marker integration, supporting both fundamental studies and drug discovery. Extending this platform to transgenic mouse models or xenograft systems would provide valuable insights into senescence in cancer and aging, and aid in the development of senescence‐targeting therapies, including senolytics.

## 4 Experimental Section

### Cell Culture and Treatments

MCF7 (HTB‐22; RRID:CVCL_0031), A549 (CCL‐185; RRID:CVCL_0006), DU145 (HTB‐81; RRID:CVCL_AS75), and WI‐38 (CCL‐75; RRID:CVCL_0579) cells were bought from American Type Culture Collection (ATCC). hTERT immortalized‐C3RO cells (referred to as C3RO hereafter) were a kind gift from Arjan Theil.^[^ ^57^ ^]^ IMR‐90 (RRID:CVCL_0347) cells were a kind gift from Joris Pothof.

MCF7, A549, WI‐38, IMR‐90 and C3RO cells were cultured in Dulbecco's Modified Eagle's Medium (DMEM, Sigma‐Aldrich, D6429, with 4.5 g L^−1^ Glucose, L‐glutamine, sodium pyruvate and sodium bicarbonate) supplemented with 10% Fetal bovine serum (FBS, Biowest, S1810) and 1% penicillin and streptomycin (Sigma‐Aldrich, P4333). DU145 cells were cultured in StableCell Roswell Park Memorial Institute medium (RPMI, Sigma‐Aldrich, R2405, with stable glutamine and sodium bicarbonate) supplemented with 10% FBS and 1% penicillin and streptomycin. Cells were grown in a 5%CO_2_/20%O_2_ 37 °C incubator and were passaged twice weekly (1:5–1:10) using Trypsin‐EDTA (TE, Sigma Aldrich, T3924). Cells were regularly tested for mycoplasma contamination (every three months) and experiments were performed with cells free of mycoplasma.

For induction of senescence, cells were seeded in 10‐cm dishes, 15‐cm dishes or 6‐well plates and incubated at 37 °C for 4–6 h. Cells were treated with 25 nM (MCF7), or 100 nM (DU145) of doxorubicin (Accord Healthcare) continuously or were given a 6 Gy or 10 Gy dose of γ‐radiation. Cells were stored at 37 °C for 4, 7 or 14 days after treatment before they were used for processing and analysis. To monitor senescence induction, 2500 cells were seeded for the untreated and 5000 cells for the doxorubicin treated conditions in each well and incubated the cells overnight. Cells were treated with 10, 25 or 50 nM doxorubicin before imaging. Cells for each condition were plated in triplicate. For induction of ER stress, cells were seeded in a confluency of 40% in a 6‐wells plate, incubated at 37 °C for 4–6 h and treated with 20 µM lipopolysaccharide (LPS, Sigma‐Aldrich, L2630) for 48 h at 37 °C.

### Reporter Cloning and Transfection

Lamin‐based reporter plasmids were derived from Dronpa‐LaminB1‐10, mCherry‐LaminA‐C‐18, pHRM‐NLS‐dCas9‐GFP11×7‐NLS‐P2A‐BFP‐dWPRE and pmScarlet‐i_C1. PiggyBac‐EGFP was a gift from Alex N. Zelensky^[^ ^58^ ^]^ and was used as initial cloning template. Dronpa‐LaminB1‐10 was a gift from Michael Davidson, used for the creation of the SRbr, SRbfr, SRsh, SRf and SRs constructs (Addgene plasmid #57 282); mCherry‐LaminA‐C‐18 was a gift from Michael Davidson (Addgene plasmid #55 068); pHRM‐NLS‐dCas9‐GFP11×7‐NLS‐P2A‐BFP‐dWPRE was a gift from Bo Huang for the creation of the SRs construct (Addgene plasmid #70 224)^[^ ^59^ ^]^; piRFP670‐N1 was a gift from Vladislav Verkhusha to create the SRbfr construct (Addgene plasmid #45 457) and pmScarlet‐i_C1 was a gift from Dorus Gadella for the SRbr, SRsh, SRf and SRs constructs (Addgene plasmid #85 044).^[^ ^60^ ^]^ pMK243 was a gift from Masato Kanemaki (Addgene plasmid #72 835)^[^ ^61^ ^]^ to create the SRsh construct. The plasmid with mCherry expressed under a human IL6‐promotor was obtained from Tebu‐bio. Plasmids were cloned using the Gibson assembly technique. plasmid DNA was stably integrated in cells using Lipofectamine 3000 Transfection reagent (Thermo Fisher Scientific, L3000015) and hyperactive PiggyBac transposase^[^ ^62^ ^]^ (8:1 ratio). Plasmid DNA was integrated in the AAVS1 safe‐harbor locus using CRISPR/Cas9 (a gift from Masato Kanemaki; Addgene plasmid # 72 833; http://n2t.net/addgene:72833; RRID:Addgene_72 833).^[^ ^61^ ^]^ Transfected cells were selected using puromycin (0.75–1.5 µg mL^−1^ (depending on construct size and cell line), InvivoGen, ant‐pr‐1).

### Proliferation Assays

Cell proliferation was studied using manual counting, EdU staining and colony formation assays. For manual counting, 1.0 × 10^6^ cells were seeded in triplicate in 10‐cm dishes. Subsequently, cells were collected from a single dish and live cell counts were conducted at 24, 48, and 72 h post‐seeding using the Countess II Automated cell counter (Invitrogen). Doubling time was calculated as follows, where growth rate is the slope of a fitted line through the growth curves on semi‐log scale (Equation 1).

(1) $$Doubling\;time=\frac{70}{growth\;rate}$$

EdU staining was performed by incorporating cells with EdU (Abcam, ab146186) in the cell culture medium to a final concentration of 30 µm for one hour at 37 °C. Cells were then fixed using 2% PFA for 15 min at room temperature and washed twice with DPBS+3% bovine serum albumin (BSA, Sigma‐Aldrich, A9418) before incubation with DPBS+0.5% Triton X‐100 (Merck Millipore, 108 603) for 20 min at room temperature. Click‐IT reaction mix (40 mM TRIS, 60 µM Atto 488 azide, 4 mM copper (II) sulphate pentahydrate and 10 mM L‐ascorbic acid) was added to the cells and incubated for 30 min at room temperature in the dark. After two washes with DPBS+3% BSA, cells were mounted using Vectashield with 4′,6‐diamidino‐2‐phenylindole (DAPI) nuclear staining (Vector Laboratories, H‐2000). EdU‐positive cell quantification was performed using the Olympus IX70 microscope.

For colony formation assays, cells (500 cells/well) were seeded in triplicate in 6‐well plates and incubated at 37 °C for 7–10 days. Colonies were fixed and stained using Coomassie Brilliant Blue solution (50% (v/v) methanol (Honeywell, 32 213), 43% (v/v) demineralized water, 7% (v/v) acetic Acid, 0.1% (w/v) Coomassie Brilliant Blue (Sigma‐Aldrich, B0149)) for 1 hour at room temperature. The number of colonies per well was counted using the Gelcount (Oxford Optronix).

### Colony Survival Assay

Colony survival assays were performed by seeding single cells (500 cells per well) in triplicate in 6‐well plates. These cells were allowed to settle overnight at 37 °C before being treated with cisplatin (0, 0.5, 1.0, 1.5, 2.0, 2.5 µm) or irradiation (0, 2, 4, 6, 8, 10 Gy). After treatment, cells were incubated at 37 °C for 7–10 days, depending on the cell line. Colonies were fixed and stained using Coomassie Brilliant Blue solution for 1 hour at room temperature. The number of colonies per well was counted using the Gelcount (Oxford Optronix).

### Immunoblotting

For immunoblotting, whole cell extracts were obtained by collecting cells in a small volume of 1xDPBS, followed by the addition of an equal volume of 2X Laemmli buffer. The samples were heated at 95 °C for 5 min and stored at −20 °C or used directly for protein quantification using Lowry assay. Subsequently, 20–50 µg of protein was loaded on a 10% SDS‐PAGE gel along with a dual‐color protein ladder (Bio‐Rad, 1 610 374). Gel electrophoresis was conducted at 50 V until the sample migrated through the stacking gel, after which the voltage was increased to 90 V until sample migration was complete. Proteins were transferred onto a FL‐PVDF membrane (Immobilon, IPFL00005) via electro‐transfer at 100 V for 1 hour.

Following transfer, the membranes were blocked using DPBS blocking solution (0.05% (v/v) Tween‐20 (Sigma‐Aldrich, P1379) +3% (w/v) skim milk powder (Sigma‐Aldrich, 70 166)) for 1 hour at 4 °C, followed by overnight incubation at 4 °C with the primary antibodies: rabbit polyclonal anti‐lamin B1 (1:2000 dilution, ab16048, Abcam); mouse monoclonal anti‐lamin A/C (1:2000 dilution, 4777, Cell Signaling Technology); rabbit polyclonal anti‐H3K9me3 (1:1000 dilution, GTX121677, GeneTex), mouse monoclonal anti‐Tubulin (1:1000 dilution, T6047, Sigma‐Aldrich) and mouse monoclonal anti‐Actin, clone C4 (1:10000 dilution, MAB1501R, Merck Millipore), all diluted in DPBS blocking solution. Subsequently, membranes were washed three times with 1xDPBS+0.05% (v/v) Tween‐20 for 10 min at room temperature, followed by 1‐hour room temperature incubation with the secondary antibodies: goat anti‐mouse IgG H+L highly cross‐adsorbed CF 680 (1:10 000 dilution, SAB4600199, Sigma‐Aldrich) and goat anti‐rabbit IgG H+L highly cross‐adsorbed CF 770 (1:10 000 dilution, SAB4600215, Sigma‐Aldrich). After another three washes, the membranes were scanned using LI‐COR's Infrared Odyssey Imaging System. Further analysis and quantification were performed with Image Studio Lite Version 5.2.

### Total RNA Isolation, cDNA Synthesis and SYBR Green qPCR

To isolate RNA, the Qiagen RNeasy mini kit was used, according to the manufacturer's protocol. Purity and concentration of isolated RNA were assessed using the NanoDrop 2000/2000c spectrophotometer. RNA samples were stored at −80 °C or used immediately for cDNA synthesis. The commercially available iScript cDNA Synthesis Kit (Bio‐Rad, 1 708 891) was used following the manufacturer's protocol. The reaction mix was incubated using the T100 Thermal Cycler from Bio‐Rad. Subsequently, purity and concentration of the synthesized cDNA was measured using the NanoDrop 2000/2000c spectrophotometer. Quantitative RT‐PCR of synthesized cDNA was performed using the commercially available IQ SYBR Green Supermix (Bio‐Rad, 1 708 882), as per the manufacturer's protocol and employing 2‐step gradient reactions. The primer sequences utilized are provided in **Table** 1.

**Table 1:** qPCR Primer sequences.

**IL6**	FW	5’ CCGGGAACGAAAGAGAAGCT 3’
	RV	5’ GCGCTTGTGGAGAAGGAGTT 3’
**GFP**	FW	5’ CGACTTCTTCAAGTCCGCCA 3’
	RV	5’ GCCGTTCTTCTGCTTGTCGG 3’
**TMEM199**	FW	5’ CACCAGCATCTGAGAGAAAGG 3’
	RV	5’ CCGTGGAGGCTTCACAAC 3’
**LMNB1**	FW	5’ AAGGCGAAGAAGAGAGGTTGAAG 3’
	RV	5’ GCGGAATGAGAGATGCTAACACT 3’
**P21**	FW	5’ GACACCACTGGAGGGTGACT 3’
	RV	5’ CAGGTCCACATGGTCTTCCT 3’
**Ki‐67**	FW	5’ TCCTTTGGTGGGCACCTAAGACCTG 3’
	RV	5’ TGATGGTTGAGGTCGTTCCTTGATG 3’
**OAZ1**	FW	5’ GGATCCTCAATAGCCACTGC 3’
	RV	5’ TACAGCAGTGGAGGGAGACC 3’
**ACTB**	FW	5’ CCAACCGCGAGAAGATGA 3’
	RV	5’ CCAGAGGCGTACAGGGATAG 3’

### FACS (Fluorescence‐Activated Cell Sorting)

Cells were seeded in 15‐cm culture dishes and treated with irradiation (6 Gy or 10 Gy) 4–6 h after seeding. Seven days later, cells were trypsinized, dissolved in cold PBS+10% FBS, and centrifuged at 1000 rpm for 5 min. The pellet was re‐dissolved in cold PBS+10%FBS and kept on ice protected from light until sorting. Sorting was based on BFP‐LMNB1 and MSC‐LMNA signal intensity, using FACS Aria II. Low intensities of BFP and mScarlet, defined by the signal of the non‐treated population, were categorized as non‐senescent. Conversely, high intensities, defined as surpassing the signal of the non‐treated population, were categorized as senescent. Cells were captured in DMEM+10%FBS+1%PS and kept on ice protected from light after sorting, for the purpose of re‐plating or isolation of protein and/or RNA.

### RNA Isolation and RNA Sequencing

Cells were seeded, treated with irradiation (6 Gy or 10 Gy), and sorted according to the procedures outlined in the “FACS” section. After sorting, total RNA was extracted using miRNeasy Mini Kit (QIAGEN, cat#217 004), incorporating a DNAse digestion on the column (QIAGEN, cat#79 254), according to manufacturer's protocol. The experiment was conducted three independent times, resulting in a total of 24 samples. The quality and concentration of total RNA were assessed using a NanoDrop 2000/2000c spectrophotometer (ThermoScientific), a Qubit 2.0 fluorometer (Invitrogen) and an Agilent 2100 BioAnalyzer, with an RNA 6000 Nano LabChip (Agilent). All RNA samples were confirmed to have a RIN score of 8.5 or higher and were stored at –80 °C before sequencing.

For sequencing, all RNA samples were diluted to 16 ng µL^−1^ using RNAse‐free water. Further preparation was conducted at the Erasmus MC Genomics Core Facility, employing a QuantSeq 3′mRNA‐SeqV2 Library Prep Kit FWD with unique dual indices (Lexogen) for library preparation, according to manufacturer's protocol. The UMI Second Strand Synthesis Module for QuantSeq FWD was used during library preparation. The resulting pool was analyzed on an Agilent Tapestation 2200 using a D1000 chip. Libraries were diluted to 2 nm and combined into a single sequencing pool. Sequencing was performed using single‐end 100 bp reads on a Nextseq2000 (Illumina).

### RNA Sequencing Analysis

Six nucleotides of the reads were extracted and appended to read names using Umi Tools v1.1.4.^[^ ^63^ ^]^ Using BBTools,^[^ ^64^ ^]^ reads aligning to Genbank U13369.1 human ribosomal DNA complete repeating unit were excluded. The alignment of samples against the human reference GRCh38 with GENCODE v38 annotation was performed using STAR v2.7.9a.^[^ ^65^ ^]^ After alignment, read groups were annotated to the aligned reads via the Add‐Or‐Replace‐Read‐Groups tool from GATK 4.3.0.0,^[^ ^66^ ^]^ followed by coordinate‐based sorting using samtools v1.17.^[^ ^67^ ^]^ Aligned reads were deduplicated using Umi Tools v1.1.4^[^ ^63^ ^]^ with the directional deduplication algorithm. Gene count matrices were generated with featureCounts v2.0.3,^[^ ^68^ ^]^ producing on average 2.7 m read counts per sample.

For downstream analysis, R v4.1.2^[^ ^69^ ^]^ was employed. DESeq2 v1.34.0^[^ ^70^ ^]^ was used for pairwise differential gene expression analysis between groups, incorporating the apeglm fold‐change shrinkage method.^[^ ^71^ ^]^ Significance was attributed to genes with an adjusted *p*‐value <0.05 and a minimum 20% change in expression (absolute log2 Fold Change ≥ log2(1.2)). Gene set enrichment analysis in GO: Biological process data, based on log2 Fold Changes in expression from selected pairwise inter‐group comparisons, was conducted using fGSEA v1.20.0.^[^ ^72^ ^]^ The assessment of changes in the expression of selected KEGG pathways was executed using pathview v1.34.0.^[^ ^73^ ^]^ Additionally, cell cycle scores of bulk RNA‐seq samples were evaluated by adapting the CellCycleScoring method from Seurat v5.0.1,^[^ ^74^ ^]^ employing the list of marker genes from Itay Tirosh et.al.^[^ ^75^ ^]^

### SA‐β‐Gal Staining

Cells were fixed and stained using the Senescence β‐galactosidase Staining Kit (Cell Signaling Technology, 9860S) following the manufacturer's protocol. Subsequently, the cells were visualized using an Olympus IX70 microscope.

### Immunofluorescence Staining

Cells were fixed with 2% PFA for 15 min at room temperature. Subsequently, cells were permeabilized and washed with 0.1% Triton X‐100 in 1xDPBS, three times short and two times for 10 min at room temperature. Following this, cells were washed once with blocking solution (1xDPBS+0.5% (w/v) BSA+0.15% (w/v) glycine (Sigma‐Aldrich, G7126)), followed by overnight incubation at 4 °C with the primary antibodies diluted in blocking solution: rabbit polyclonal anti‐histone H3K9me3 (1:500 dilution, GTX121677, GeneTex); rabbit polyclonal anti‐lamin B1 (1:200 dilution, ab16048, Abcam); mouse monoclonal anti‐lamin A/C (1:100 dilution, 4777, Cell Signaling Technology); rabbit polyclonal anti‐53BP1 (1:1000 dilution, NB100‐904, Novus Biologicals); mouse monoclonal anti‐phospho Histone H2A.X (Ser139), clone JBW301 (1:1000 dilution, 05–636, Merck Millipore) and rabbit polyclonal anti‐Geminin (1:400 dilution, 10802‐1‐AP, Proteintech). Washing steps with 0.1% (v/v) Triton X‐100 were repeated, followed by a 1 h incubation at room temperature incubation (dark) with the secondary antibodies: goat anti‐rabbit Alexa Fluor 488 IgG H+L (1:1000 dilution, ab150077); goat anti‐mouse Alexa Fluor 488 IgG H+L (1:1000 dilution, ab150113) and goat anti‐rabbit Alexa Fluor 555 IgG H+L (1:1000 dilution, ab150078) (Abcam). Further washing with 0.1% Triton X‐100 in 1xDPBS was performed and cells were mounted using Vectashield mounting medium with or without 4,6‐diamidino‐2‐phenylindole (DAPI) staining (Vector Laboratories, H‐(1)/(2)000).

### Confocal Microscopy

Cells were imaged using either the SP5 or SP8 confocal microscope from Leica Microsystems. For imaging of 2D monolayers, Leica's HC PL APO 40X/1.30 OIL CS2 objective on the SP8 and the HCX PL APO 40X/1.25 OIL CS objective on the SP5 microscope were used. Sequential scanning was employed to prevent crosstalk when detecting multiple fluorophores in a single sample. The following excitation and filter settings were applied: 405 nm excitation and 415–470 nm for detection of mTagBFP2 or DAPI nuclear stain, 488 nm excitation and 500–550 nm for detection of EGFP or Alexa Fluor 488, 561 nm excitation and 570–620 nm for detection of mScarlet‐I, 633 nm excitation and 645–750 nm for detection of Alexa Fluor 647.

### High content Screening

For monitoring senescence induction with doxorubicin, MCF7 cells transfected with SRbfr and NLS‐mScarlet were imaged with the Opera Phenix HCS microscope over 7 days. Images were acquired by a 20X objective at 12 h intervals. Laser excitation wavelengths were: 405 nm (blue channel: BFP‐laminB1), 561 nm (red channel: NLS‐mScarlet), and 640 nm (far‐red channel: RFP670‐laminA).

### Imaging Analysis

For the analysis of confocal images, an image analysis pipeline was developed in Fiji^[^ ^76^ ^]^ to measure the mean intensities of transgenic lamins, antibody staining and fluorescent proteins in nuclei from single cells. In brief, obtained confocal images (lif files; Leica) were opened in Fiji using the Bioformats plugin.^[^ ^77^ ^]^ The images were split into separate channels, one channel for each color. Images of the lamin A intensity were segmented to obtain regions of interest (ROIs). First a Gaussian blur was applied, followed by a Li‐threshold^[^ ^78^ ^]^ and a Laplacian‐of‐Gaussian kernel. The resulting images were used to detect local maxima and to segment the nuclei into ROIs. For each obtained ROI, the mean intensities of the protein of interest were determined. The obtained mean intensities were exported to Excel files. Based on these intensities of the protein of interest the threshold for positivity: intensity> the mean of intensities +2*standard deviation of the control (d4‐) was determined.

The 53BP1 and γH2AX foci were quantified using the Image‐J macro “Foci‐analyzer” (freely available at https://github.com/BioImaging‐NKI/Foci‐analyzer; developed by Bram van den Broek, the Netherlands Cancer Institute, the Netherlands).^[^ ^79^ ^]^

### Senescence Screening Analysis

The Excel files generated by the imaging analysis program were imported into Matlab (Mathworks, R2023b) and analyzed using a senescence screening analysis program. In brief, the program determines the linear correlation between the mean transgenic lamin B1‐ and lamin A intensities of the control condition for each experiment (day 4 untreated: d4‐). The program considers cells as senescent based on their deviation from this linear correlation and the lamin intensities. The measure of deviation relies on the 95% confidence interval of the linear correlation between the B1 and A lamin intensities in the control. For every treatment condition, each individual cell is categorized as senescent or non‐senescent. The program utilizes R‐squared as a measure of heterogeneity within the cell population and implements correction of the used parameters if R^2^<0.95. The same parameters for determining the measure of deviation to categorize the cells were used for each experiment and each cell line. The linear correlation and 95% confidence interval are determined for each individual experiment based on the control condition. The accuracy of our screening method was calculated using the true positives (TP), true negatives (TN), false positives (FP) and false negatives (FN) (Equation 2).

(2) $$Accuracy=\frac{TN+TP}{TN+TP+FP+FN}$$

For senescence analysis of the high‐content screening images, their own control was used for each well: the first image (t = 0 h) instead of comparing treated versus untreated wells.

### Statistical Analysis

Data was normalized to determine fold‐changes were applicable. Data in graphs is presented as the mean with error bars showing standard deviation. Statistical analysis was conducted using at least three independent experiments with Student's *t*‐tests (unpaired, parametric two‐tailed tests with a 95% confidence interval) or one/two‐way ANOVA tests in GraphPad Prism v10.2.1 (GraphPad Software Inc.). When Student's *t*‐tests were performed, data was assumed to follow a normal distribution. Equal variance was tested by performing F‐tests. Differences were considered non‐significant when *p* > 0.05 and significant when * *p* < 0.05; ** *p* < 0.01; *** *p* < 0.001; and **** *p* < 0.0001.

## Conflict of Interest

The authors declare no conflict of interest.

## Supporting information



Supporting Information

## Data Availability

The data that support the findings of this study are available from the corresponding author upon reasonable request.
