# Supplementary material for: A Reporter Platform to Study Therapy‐Induced Senescence in Live Cancer Cells
Source: Small Methods. 2025 Oct 29;9(12):e01270. doi: 10.1002/smtd.202501270 (PMC12716209; doi:10.1002/smtd.202501270)
Supplement: Supplementary file 1 — Supporting Information [file SMTD-9-e01270-s001.docx]

Supporting Information

A Reporter Platform to Study Therapy-induced Senescence in Live Cancer Cells

*Jacinta van de Grint^1,2^, Mengqi Huang^3^, Ruben Sangers^1^, Hanny Odijk^1^, Thom Reuvers^1^, Jose M. Heredia-Genestar^1^, Anja Raams^1^, Tsung Wai Kan^3^, Joris Pothof^1^, Roland Kanaar^1,2^ and Maayke M.P. Kuijten^1,2,*^*

J. van de Grint, R. Sangers, H. Odijk, T. Reuvers, J.M. Heredia-Genestar, A. Raams, J. Pothof, R. Kanaar, M.M.P. Kuijten

Department of Molecular Genetics, Oncode Institute, Erasmus MC Cancer Institute, Erasmus University Medical Center, Rotterdam, 3015 GD, the Netherlands

M. Huang, T. W. Kan

Department of Pathology, Erasmus University Medical Center, Rotterdam, 3015 GD, the Netherlands

* Corresponding author:

Maayke M.P. Kuijten. E-mail: m.kuijten@erasmusmc.nl


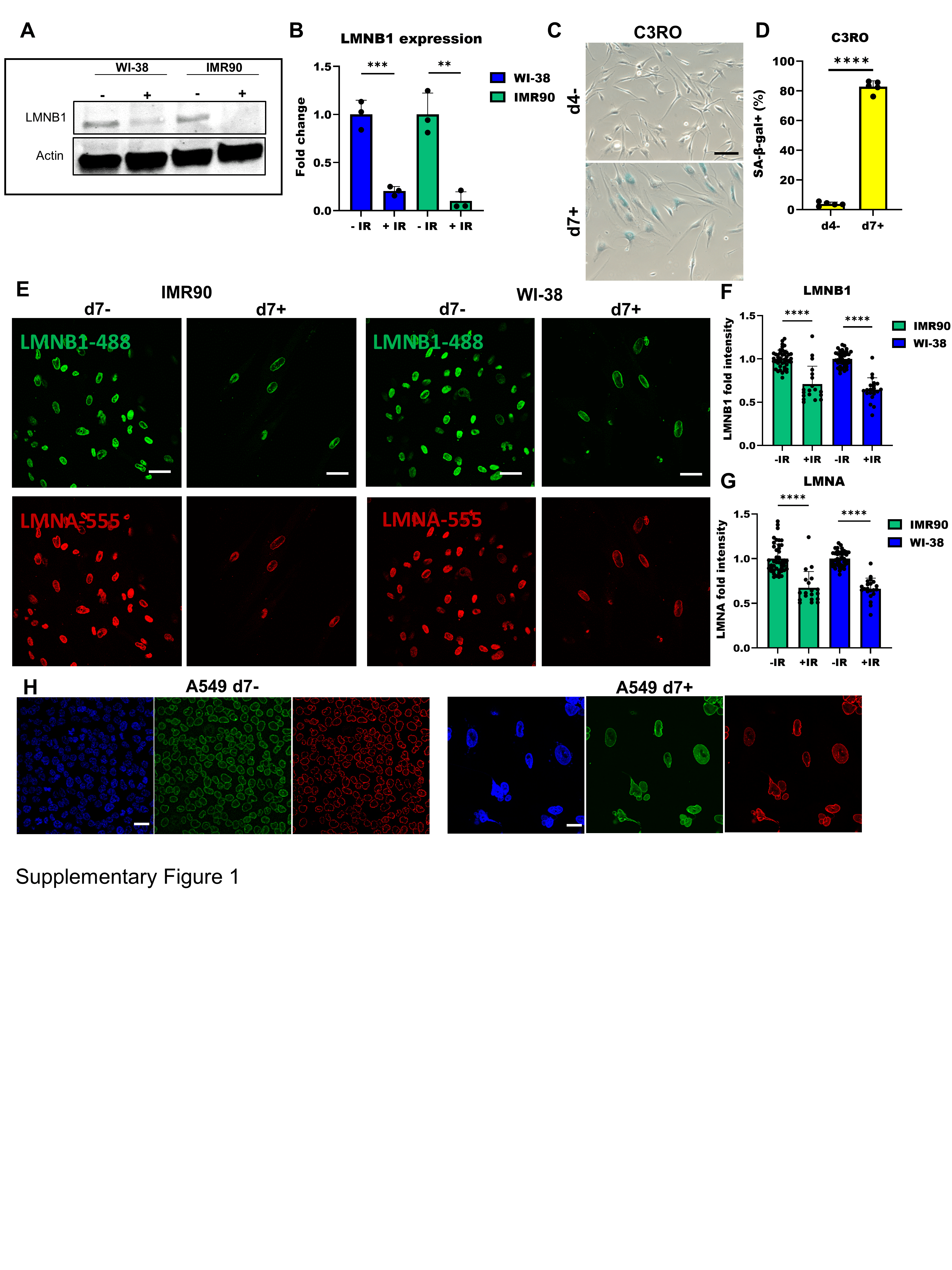


**Figure S1: Therapy-induced senescence in cancer cells and human diploid fibroblasts. A.** Immunoblot of WI-38 and IMR90 cells for LMNB1 (endogenous), 7 days post seeding (non-treated, d7-) and 7 days post treatment (10 Gy, d7+). Loading control: Actin. **B.** Quantification of LMNB1 expression on day 7 in WI-38 and IMR90 cells, untreated or treated with 10 Gy. **C.** Representative SA-β-Gal images of C3RO cells, untreated (d4-) or treated with 75 nM doxorubicin (d7). Scale bar = 100 µm. **D.** Percentage of SA-β-Gal+ C3RO cells, untreated (d4-) or treated with 75 nM doxorubicin (d7+). **E.** Confocal images of IMR90 and WI-38 cells, untreated or 10 Gy-treated, stained for LMNB1 (green) and LMNA (red) on day 7. Scale bar = 50 µm. **F.** Quantification of LMNB1 staining signal in IMR90 and WI-38 cells. **G.** Quantification of LMNA staining signal in IMR90 and WI-38 cells. **H.** Confocal images of A549 cells, stained for DAPI, LMNA (endogenous), and LMNB1 (endogenous), 4 and 7 days post seeding (non-treated, d4- and d7-) and 7 days post treatment (10 Gy, d7+). Scale bar: 25 µm. B, D, F and G, Data was presented with mean and error bars showing standard deviation. Statistics: unpaired parametric two-tailed Student’s t-tests; ** *P* <0.01; *** *P* <0.001; **** *P* <0.0001.


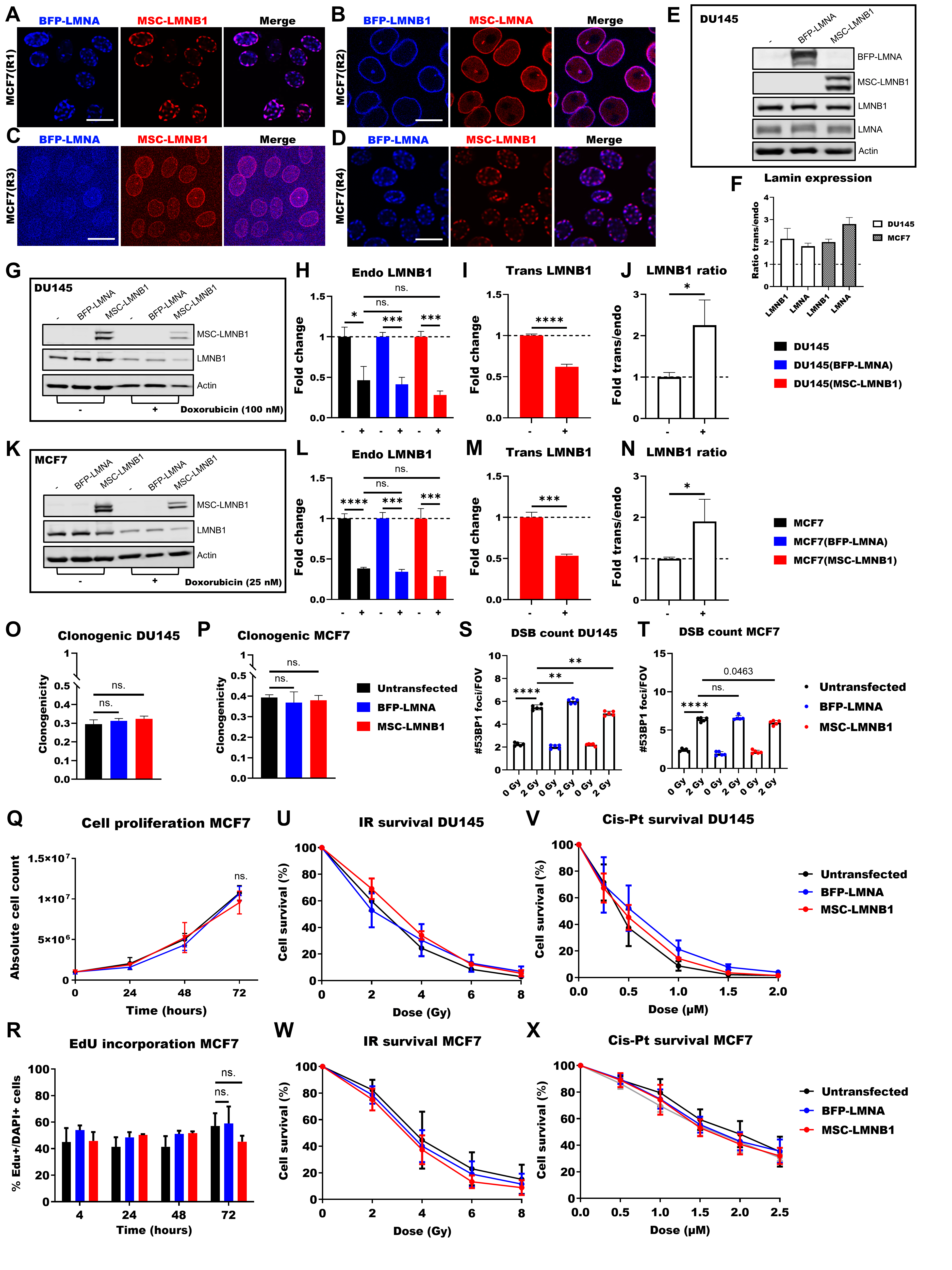


**Figure S2: Development of the senescence reporter.** **A**. MCF7 cells transfected with reporter candidate 1 (R1). Scale bar: 25 µm. **B**. MCF7 cells transfected with R2. **C**. MCF7 cells transfected with R3. **D**. MCF7 cells transfected with R4. **E**. Representative immunoblot images from DU145 cells expressing BFP-LMNA or MSC-LMNB1. Loading control: Actin. Protein bands are in original order. **F**. Ratio calculation between MSC-LMNB1 and endogenous LMNB1 in DU145 and MCF7 cells (n=3). **G.** Representative immunoblot images from DU145 cells expressing no construct, BFP-LMNA or MSC-LMNB1, untreated or doxorubicin-treated 7 days post-treatment. Loading control: Actin. Protein bands are in original order. **H.** Quantification of immunoblot for endogenous LMNB1 in DU145 cells corresponding to panel G (n=3). **I.** Quantification of immunoblot for MSC-LMNB1 in DU145 cells expressing MSC-LMNB1 (n=3). **J.** Fold ratio calculation between MSC-LMNB1 and endogenous LMNB1 in DU145 cells (n=3). **K.** Representative immunoblot images from MCF7 cells expressing no construct, BFP-LMNA or MSC-LMNB1, untreated or doxorubicin-treated 7 days post-treatment. Loading control: Actin. Protein bands are in original order. **L.** Quantification of immunoblot for endogenous LMNB1 in MCF7 cells corresponding to panel K (n=3). **M.** Quantification of immunoblot for MSC-LMNB1 in MCF7 cells expressing MSC-LMNB1 (n=3). **N.** Fold ratio calculation between MSC-LMNB1 and endogenous LMNB1 in MCF7 cells (n=3). **O.** Clonogenicity calculation from DU145 cells expressing no construct, BFP-LMNA or MSC-LMNB1 (n=3). **P.** Clonogenicity calculation from MCF7 cells expressing no construct, BFP-LMNA or MSC-LMNB1. **Q.** Growth curve of MCF7 cells in culture expressing no construct, BFP-LMNA or MSC-LMNB1, measured by absolute cell numbers at 24, 48 and 72 hours after seeding, respectively (n=3). **R.** EdU incorporation 4, 24, 48, and 72 hours after seeding, to quantify the amount of S-phase cells. MCF7 cells expressing no construct, BFP-LMNA or MSC-LMNB1 (n=3). **S.** Quantification of the number of 53BP1 foci calculated per cell, untreated and 2 Gy treated 2 hours post-treatment. DU145 cells used expressing no construct, BFP-LMNA or MSC-LMNB1. **T.** Quantification of the amount of 53BP1 foci calculated per cell, untreated and 2 Gy treated 2 hours post-treatment. MCF7 cells used expressing no construct, BFP-LMNA or MSC-LMNB1.

**U.** Colony survival assay (7 days) using multiple doses of irradiation on DU145 cells expressing no construct, BFP-LMNA or MSC-LMNB1 (n=3). **V.** Colony survival assay (7 days) using cisplatin on DU145 cells expressing no construct, BFP-LMNA or MSC-LMNB1 (n=3). **W.** Colony survival assay (7 days) using irradiation on MCF7 cells expressing no construct, BFP-LMNA or MSC-LMNB1 (n=3). **X**. Colony survival assay (7 days) using cisplatin on MCF7 cells expressing no construct, BFP-LMNA or MSC-LMNB1 (n=3).

F, H-J, L-N, O-X, Data was presented with mean and error bars showing standard deviation. Statistics: unpaired parametric two-tailed Student’s t-tests; ns. *P* >0.05; * *P* <0.05; ** *P* <0.01; *** *P* <0.001; **** *P* <0.0001.

**
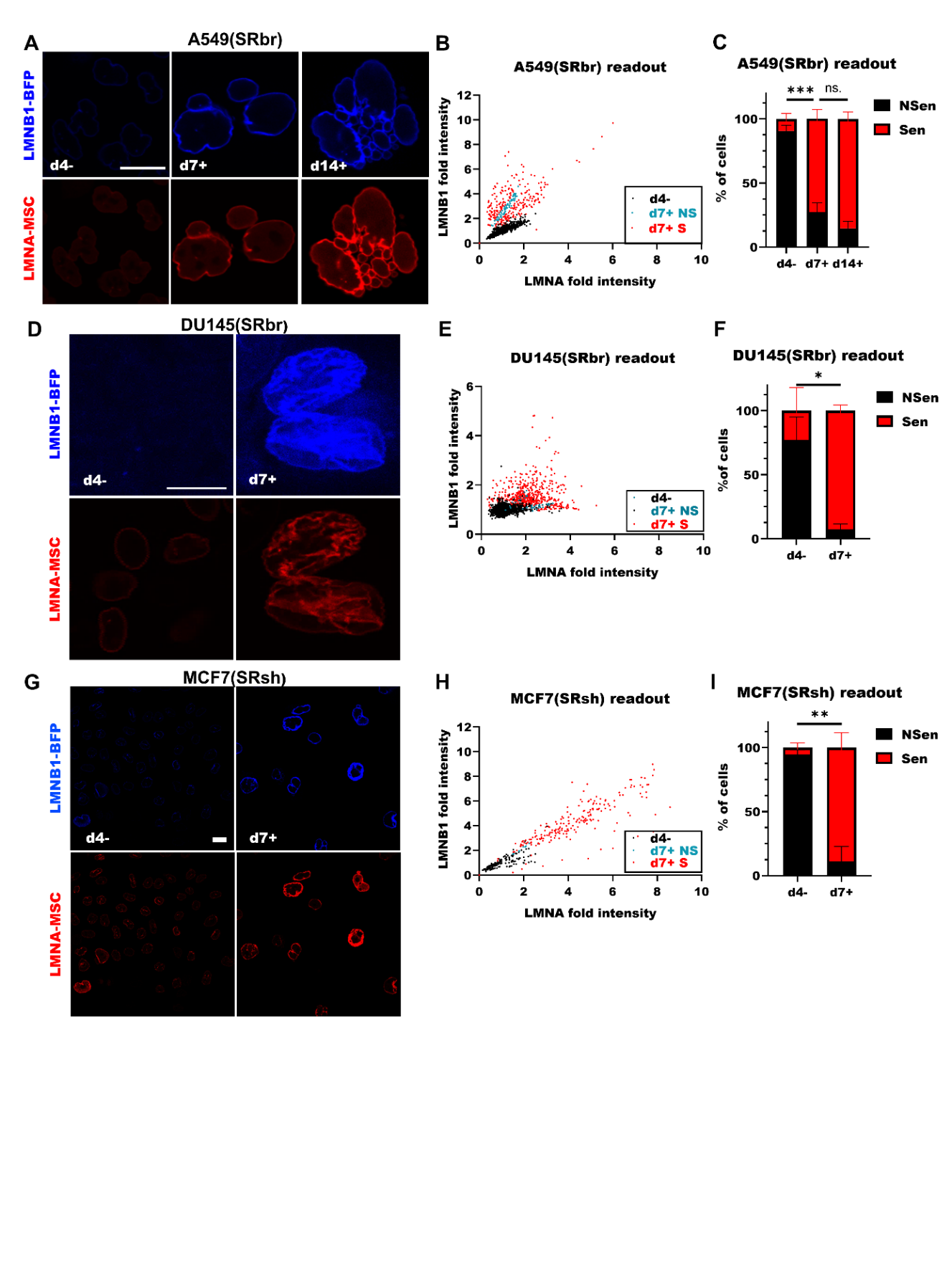
**

**Figure S3: Analysis of senescence in cancer cells.** **A.** Confocal images of transgenic LMNB1-BFP and LMNA-MSC in untreated (d4-) and 10 Gy-treated A549(SRbr) cells 7 and 14 days post-treatment (d7+ and d14+). Scale bar: 25 µm. **B.** Scatterplot of A549(SRbr) cells, 4 days post seeding (non-treated, d4-) and 7 days post treatment (10 Gy, d7+). **C.** Number of senescent and non-senescent cells in untreated and 10 Gy-treated A549(SRbr) cells determined with screening assay (n=3). **D.** Confocal images of transgenic LMNB1-BFP and LMNA-MSC in untreated (d4-) and 10 Gy-treated DU145(SRbr) cells 7 days post-treatment (d7+). Scale bar: 25 µm. **E.** Scatterplot of DU145(SRbr) cells, 4 days post seeding (non-treated, d4-) and 7 days post treatment (10 Gy, d7+). **F.** Number of senescent and non-senescent cells in untreated and 10 Gy-treated DU145(SRbr) cells determined with screening assay (n=3). **G.** Confocal images of transgenic LMNB1-BFP and LMNA-MSC in untreated (d4-) and 10 Gy-treated MCF7(SRsh) cells, 7 days post treatment (d7+). Scale bar: 25 µm. **H.** Scatterplot of MCF7(SRsh) cells, 4 days post seeding (non-treated, d4-) and 7 days post treatment (10 Gy, d7+).

**I.** Quantification of the number of senescent and non-senescent cells in untreated and 10 Gy-treated MCF7(SRsh) cells determined with screening assay (n=3). C, F and I, Data was presented with mean and error bars showing standard deviation. Statistics: unpaired parametric two-tailed Student’s t-tests; ns. *P* >0.05; * *P* <0.05; ** *P* <0.01; *** *P* <0.001.

**
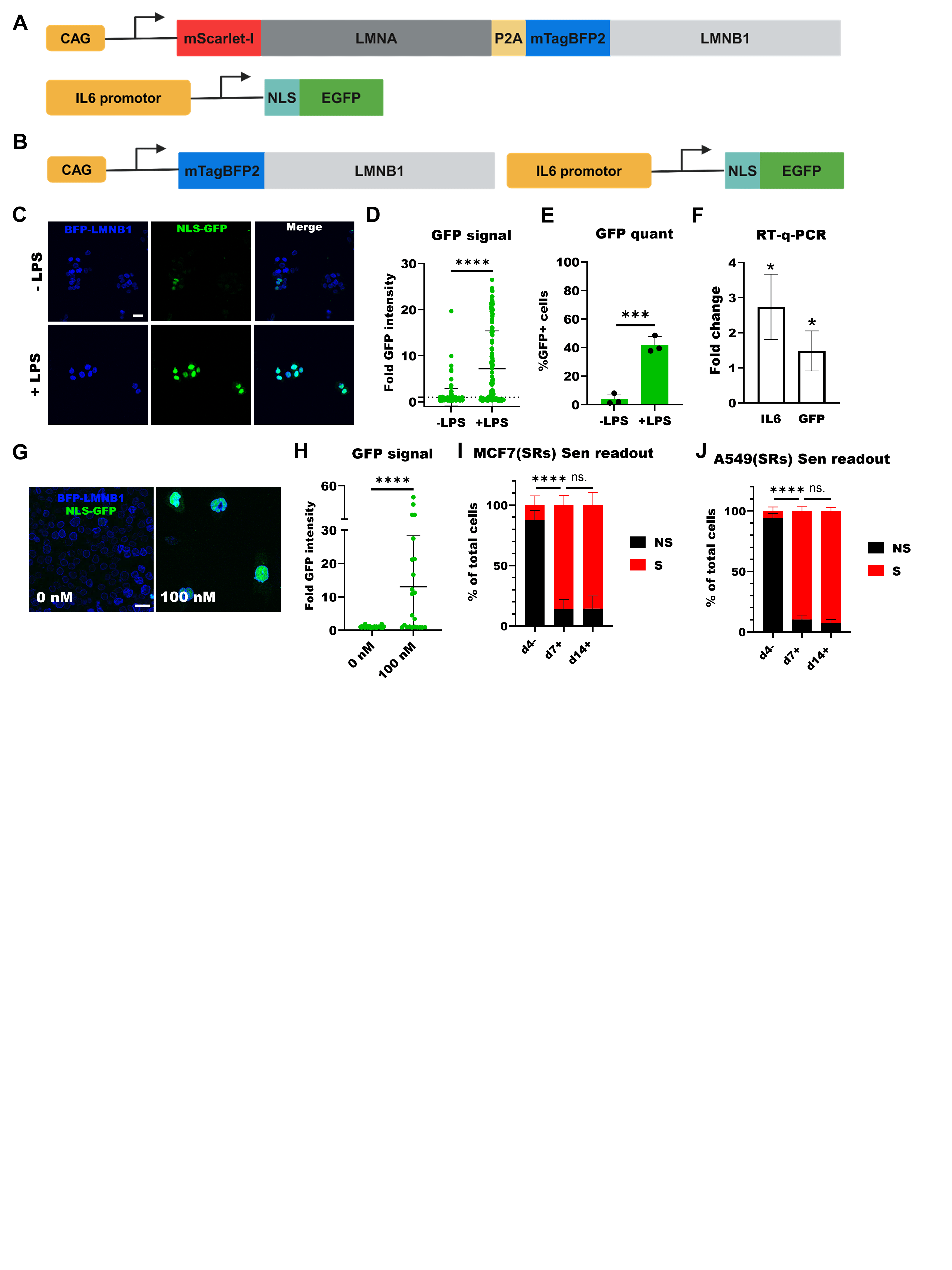
**

**Figure S4: IL6 upregulation in senescent cancer cells.** **A.** Construct with LMNB1 and LMNA fused to mScarlet-I (MSC) or mTagBFP2 (BFP) for labeling nuclear lamins. Additional IL6-promotor, regulating the transcription of NLS-EGFP (NLS-GFP). CAG: CAG-promotor; P2A: P2A-linker; NLS: Nuclear localization signal. **B.** Construct with LMNB1 fused to mTagBFP2 (BFP) for labeling nuclear lamins. Additional IL6-promotor, regulating the transcription of NLS-EGFP (NLS-GFP). **C.** Confocal images of untreated and lipopolysaccharide (LPS)-treated DU145(IL6REP) cells 48 hours post-treatment. Scale bar: 25 µm.

**D.** Normalized fold intensity of GFP signal in untreated compared to LPS-treated cells. **E.** Percentage of GFP+ cells in the untreated compared to LPS-treated population. **F.** Normalized fold change from RT-qPCR analysis of untreated compared to LPS-treated cells (n=3). **G.** Confocal images of untreated and doxorubicin-treated DU145(IL6RT) cells 7 days post-treatment. Scale bar: 25 µm. **H.** Normalized fold intensity of GFP signal in untreated compared to doxorubicin-treated cells. **I.** Percentage of senescent and non-senescent MCF7(SRs) cells in untreated (d4-) and 10 Gy-treated cells (d7+ and d14+) determined with screening assay (n=3). **J.** Percentage of senescent and non-senescent A549(SRs) cells (n=3). D-F and H-J, Data was presented with mean and error bars showing standard deviation. Statistics: unpaired parametric two-tailed Student’s t-tests; ns. *P* >0.05; * *P* <0.05; *** *P* <0.001; **** *P* <0.0001.

**
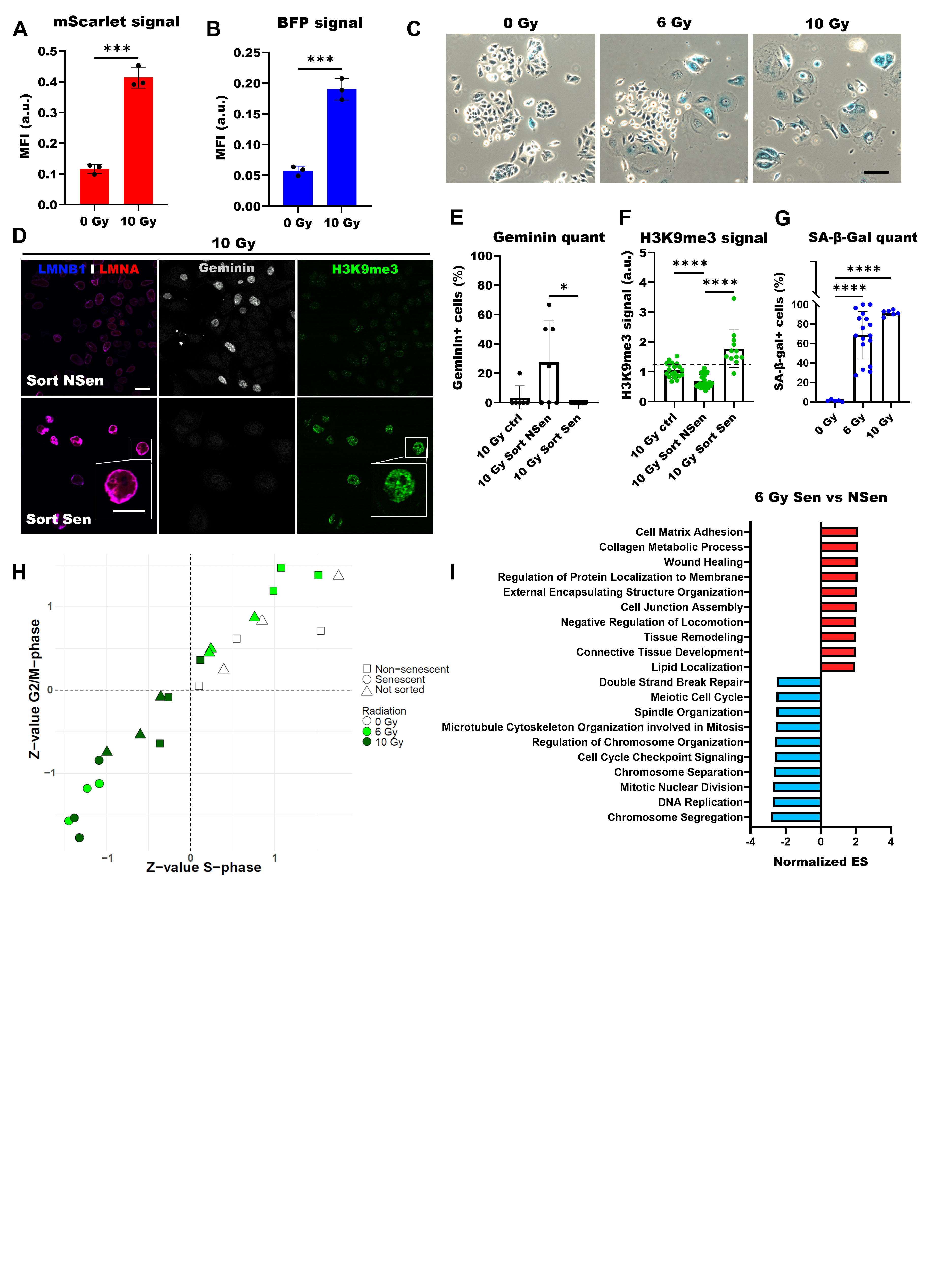
**

**Figure S5: Sorting senescent versus non-senescent cells.**

**A.** Quantification of the MSC mean fluorescence intensity (MFI) in untreated and 10 Gy-treated MCF7(SRbr) cells, 7 days post-treatment. **B.** Quantification of the BFP mean fluorescence intensity (MFI). **C.** Untreated (0 Gy), 6 Gy (6 Gy) and 10 Gy-treated (10 Gy) cells, 7 days post-treatment, stained for SA-β-Gal. Scale bar: 100 µm. **D.** Confocal images of 10 Gy-treated, sorted MCF7(SRbr) cells, categorized as non-senescent (Sort NSen) versus senescent (Sort Sen) based on LMNA-MSC and LMNB1-BFP intensities 7 days after treatment, co-stained for geminin and H3K9me3. Scale bar: 25 µm. **E.** Percentage of geminin+ cells of 10 Gy-treated, sorted MCF7(SRbr) cells. **F.** Normalized intensity of H3K9me3 signal in 10 Gy-treated, sorted MCF7(SRbr) cells.

**G.** Percentage of SA-β-Gal+ MCF7(SRbr) cells 7 days post-treatment corresponding to panel C. **H.** Cell cycle Z-scores for G2/M-phase and S-phase of all RNA-seq samples (n=3), evaluated by adapting the Cell Cycle Scoring method from Seurat v5.0.1, reflecting the relative probability that cells were in G2/M or S-phase of the cell cycle. **I.** Pathway enrichment analysis of 6 Gy-treated, sorted and categorized as senescent vs non-senescent populations. Normalized enrichment score (NES): the over-representation of this gene set in the list of differentially expressed genes, normalized across the analyzed gene sets. *Unt = untreated, Ctrl = not sorted, Sort NSen = sorted, categorized as non-senescent, Sort Sen = sorted, categorized as senescent based on transgenic LMNA and LMNB1 intensity.* A-B and E-G, Data was presented with mean and error bars showing standard deviation. Statistics: unpaired parametric two-tailed Student’s t-tests; * *P* <0.05; *** *P* <0.001; **** *P* <0.0001.


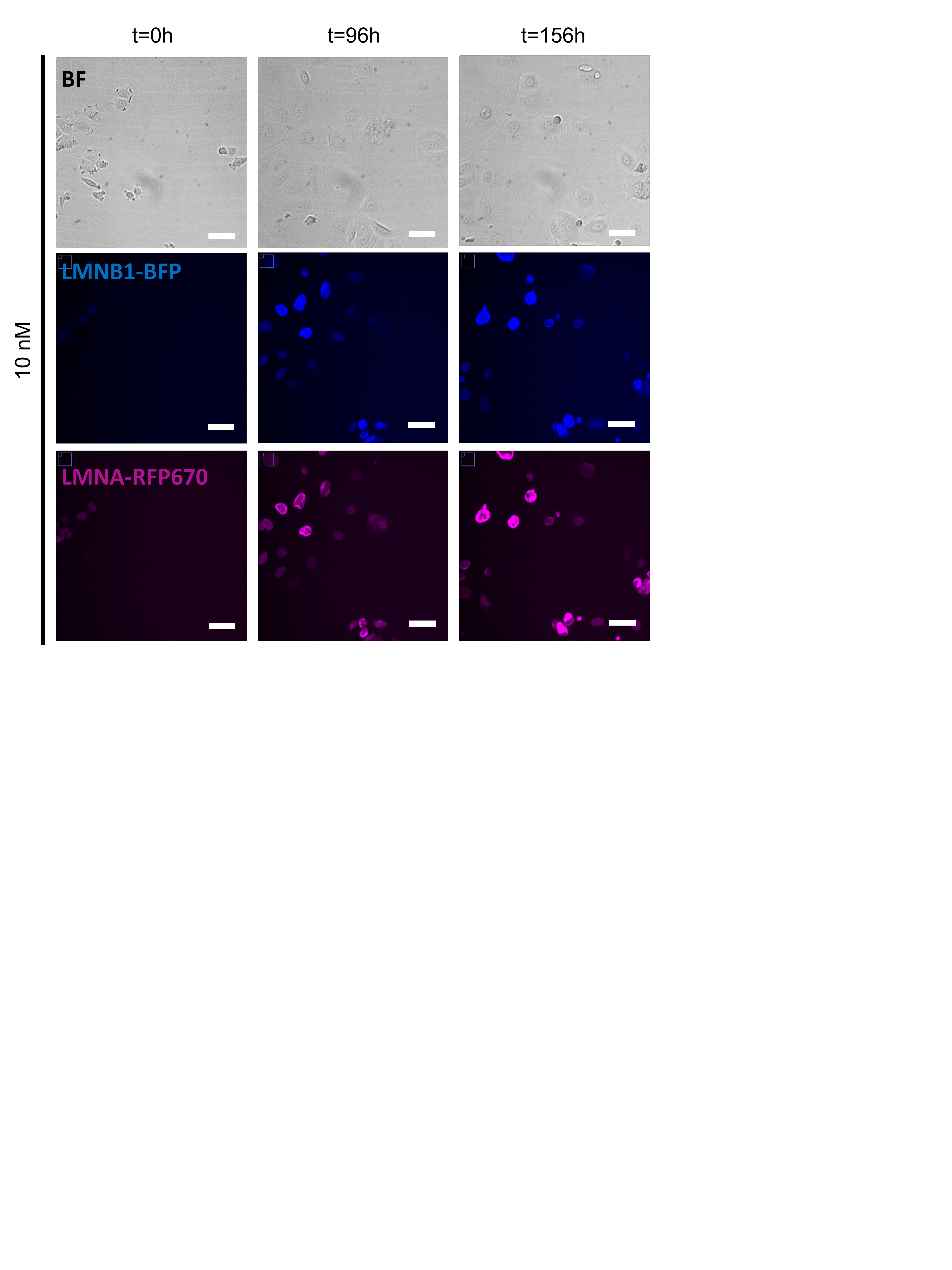


**Figure S6: Monitoring senescence induction.**

Time-lapse images of doxorubicin-treated (10 and 25 nM) MCF7(SRbfr) cells, imaged in the bright-field channel (BF), LMNB1-BFP (blue), and LMNA-RFP670 (pink) channel. Three time points are shown: 0 h, 84 h and 156 h. Scale bar = 50 µm.

**
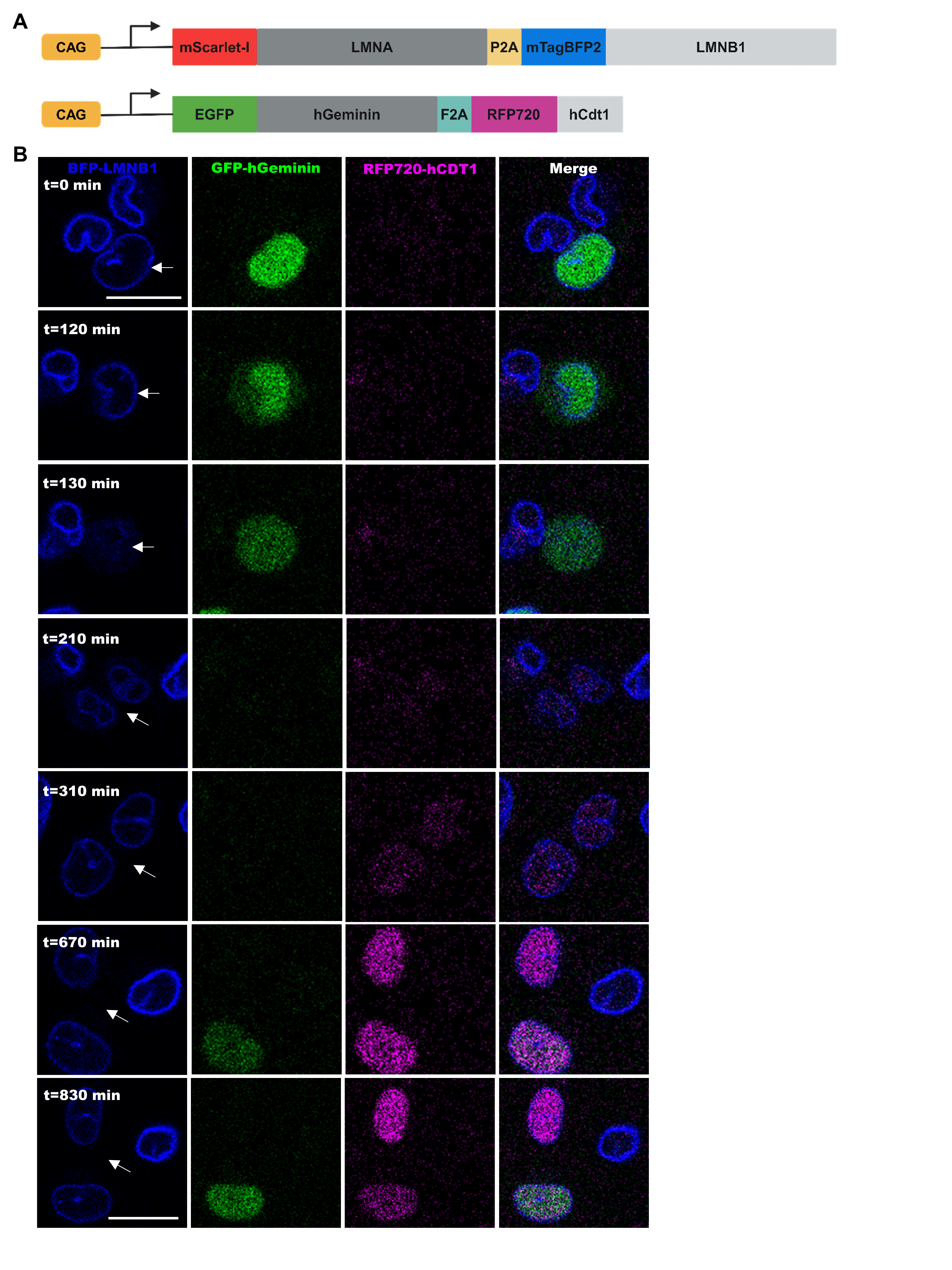
**

**Figure S7: Monitoring senescence induction and escape.** **A.** Construct with LMNB1 and LMNA fused to mTagBFP2 (BFP) or mScarlet-I (MSC) for labeling nuclear lamins. Construct with hGeminin and hCdt1 fused to EGFP and iRFP720 respectively. CAG: CAG-promotor; P2A: P2A-linker; F2A: F2A-linker. **B.** Confocal time-lapse (0–700 min) of MCF7(SRf) cells, monitoring LMNB1-BFP, LMNA-MSC, GFP-geminin and RFP670-CDT1.
